# miR-31-5p regulates cold acclimation of the wood-boring beetle *Monochamus alternatus* via ascaroside signaling

**DOI:** 10.1186/s12915-020-00926-w

**Published:** 2020-11-27

**Authors:** Bin Zhang, Lilin Zhao, Jing Ning, Jacob D. Wickham, Haokai Tian, Xiaoming Zhang, Meiling Yang, Xiangming Wang, Jianghua Sun

**Affiliations:** 1 State Key Laboratory of Integrated Management of Pest Insects and Rodents, Institute of Zoology, Chinese Academy of Sciences, Beijing, 100101 China; 2 CAS Center for Excellence in Biotic Interactions, University of Chinese Academy of Sciences, Beijing, 10049 China; 3 National Laboratory of Biomacromolecules, Institute of Biophysics, Chinese Academy of Sciences, Beijing, China

**Keywords:** Ascaroside, miRNAs, Phenotypic plasticity, Cold acclimation, *Monochamus alternatus*

## Abstract

**Background:**

Survival to cold stress in insects living in temperate environments requires the deployment of strategies that lead to physiological changes involved in freeze tolerance or freeze avoidance. These strategies may consist of, for instance, the induction of metabolic depression, accumulation of cryoprotectants, or the production of antifreeze proteins, however, little is known about the way such mechanisms are regulated and the signals involved in their activation. Ascarosides are signaling molecules usually known to regulate nematode behavior and development, whose expression was recently found to relate to thermal plasticity in the Japanese pine sawyer beetle *Monochamus alternatus*. Accumulating evidence also points to miRNAs as another class of regulators differentially expressed in response to cold stress, which are predicted to target genes involved in cold adaptation of insects. Here, we demonstrate a novel pathway involved in insect cold acclimation, through miRNA-mediated regulation of ascaroside function.

**Results:**

We initially discovered that experimental cold acclimation can enhance the beetle’s cold hardiness. Through screening and functional verification, we found miR-31-5p, upregulated under cold stress, significantly contributes to this enhancement. Mechanistically, miR-31-5p promotes production of an ascaroside (asc-C9) in the beetle by negatively targeting the rate-limiting enzyme, acyl-CoA oxidase in peroxisomal β-oxidation cycles. Feeding experiments with synthetic asc-C9 suggests it may serve as a signal to promote cold acclimation through metabolic depression and accumulation of cryoprotectants with specific gene expression patterns.

**Conclusions:**

Our results point to important roles of miRNA-mediated regulation of ascaroside function in insect cold adaptation. This enhanced cold tolerance may allow higher survival of *M. alternatus* in winter and be pivotal in shaping its wide distribution range, greatly expanding the threat of pine wilt disease, and thus can also inspire the development of ascaroside-based pest management strategies.

**Supplementary information:**

The online version contains supplementary material available at 10.1186/s12915-020-00926-w.

## Background

Low temperatures pose a major challenge for insects in a highly seasonal temperate zone environment. To survive cold winters, insects have evolved strategies such as freeze tolerance by having the ability to survive internal ice formation and freeze avoidance by supercooling [1–4]. These strategies require diverse physiological changes including metabolic depression, accumulation of cryoprotectants, ion and water balance, and production of antifreeze proteins [4–7]. While much is now known about physiological plasticity involved in insect cold hardiness, signaling processes involving the acquisition of cold tolerance are still poorly understood.

As autumn progresses, insects respond to environmental signals such as shortening day length and decreasing temperatures with dramatic physiological changes that trigger seasonal cold acclimation and initiation of diapause [3, 6]. Signaling pathways involving hormonal control [8], energetic regulation [9], and insulin signaling [10, 11], which are associated with entry into diapause, have received much attention. As diapause and cold tolerance are often inextricably linked, these signaling pathways also provide some insights for insect cold tolerance. Mechanistically, insects first sense the cold signals, then activate the signaling pathways and reprogram the expression of cold-responsive genes that ultimately leads to metabolite changes [12, 13]. Sometimes, these thermal-dependent metabolites are not only a passive target of cold signaling, but could also act as a secondary chemical signal that functions like hormones to initiate cold signaling and ultimately regulates cold-responsive gene expression [9, 14]. Although the cryoprotective roles of these metabolites in insect cold adaptation have been widely recognized [6, 15, 16], our understanding of their potential roles in signaling is still limited.

Cold stress-relevant changes in gene expression have been documented for a variety of insect species such as bumble bees, mosquitoes, and moths [17–19]. However, their epigenetic regulatory networks remain largely unknown. MicroRNA (miRNA), small non-coding RNAs of ∼ 21 to 24 nucleotides in length, are a ubiquitous epigenetic regulator at the post-transcriptional level in organisms. Accumulating evidence from sequencing data have also uncovered several subsets of miRNAs that are differentially expressed in response to cold stress and are predicted to target some critical genes for physiological changes, suggesting miRNAs are potentially involved in cold adaptation in insects [20–22], while the mechanism by which miRNAs regulate cold adaptation remains unexplored.

The Japanese pine sawyer beetle, *Monochamus alternatus* Hope (Coleoptera: Cerambycidae), is not only a serious wood-boring pest in itself, but is also the main vector of the invasive pinewood nematode (PWN), *Bursaphelenchus xylophilus*, which is native to North America and causes destructive pine wilt disease over a wide temperate range including Japan, Korea, China, and Europe [23]. This beetle is a freeze-avoiding insect and has developed several mechanisms such as facultative diapause, pre-emptive acclimation, and seasonal and developmental variation in cold hardiness to mitigate cold winters [24, 25]. During both natural and experimental cold acclimation, supercooling point (SCP), which is the lowest temperature at which an abrupt rebound on the thermal curve occurs due to the release of the latent heat of ice crystallization, significantly drops and survival rate increases in this beetle, indicating enhancement of cold hardiness [24], but mechanisms underlying this process have not been well explored.

Intriguingly, ascarosides, widely conserved small signaling molecules in nematodes that contain dideoxy sugar ascarylose and a fatty acid side chain [26], were also found to be produced by this beetle [23]. Moreover, the larval beetle, which is unable to vector PWN, produced asc-C9 that harbors 9 carbons on the side chain and is known as ascr#10 in nematodes [27], and it was shown this ascaroside retards the larva’s development to synchronize its life cycle with the nematodes its adult vectors, maintaining their mutualistic relationship [23]. It is noteworthy that abundance of this small molecule correlated positively with low temperature [23]: (i) In the field, asc-C9 is mostly produced by the beetle during the last larval instar, which experiences low-temperature stress during cold winters; (ii) in the laboratory, the amount of asc-C9 increases during an experimental cold acclimation process (4 °C). This correlation between ascaroside abundance and low temperature thus suggests a potential role of ascarosides in cold acclimation of this beetle.

Here, we hypothesized that miRNAs and ascarosides might be involved in insect cold signaling. To test this hypothesis, we first demonstrated cold acclimation can enhance the beetle’s cold hardiness. Subsequently, we screened miR-31-5p, a miRNA whose expression was upregulated under cold stress, and validated that it significantly facilitates the observed cold tolerance. Mechanistically, miR-31-5p negatively targets the rate-limiting enzyme, acyl-CoA oxidase (ACOX1), in the peroxisomal β-oxidation cycle that shortens fatty acid chains of ascarosides. Feeding experiments with synthetic asc-C9 and gene expression analysis suggested the ascaroside might act as a signal to regulate the beetle’s cold tolerance by metabolic suppression and cryoprotectant accumulation. These results demonstrate the important roles of miRNAs and ascarosides in insect cold acclimation.

## Results

### Cold acclimation enhances cold hardiness

**Fig. 1:**
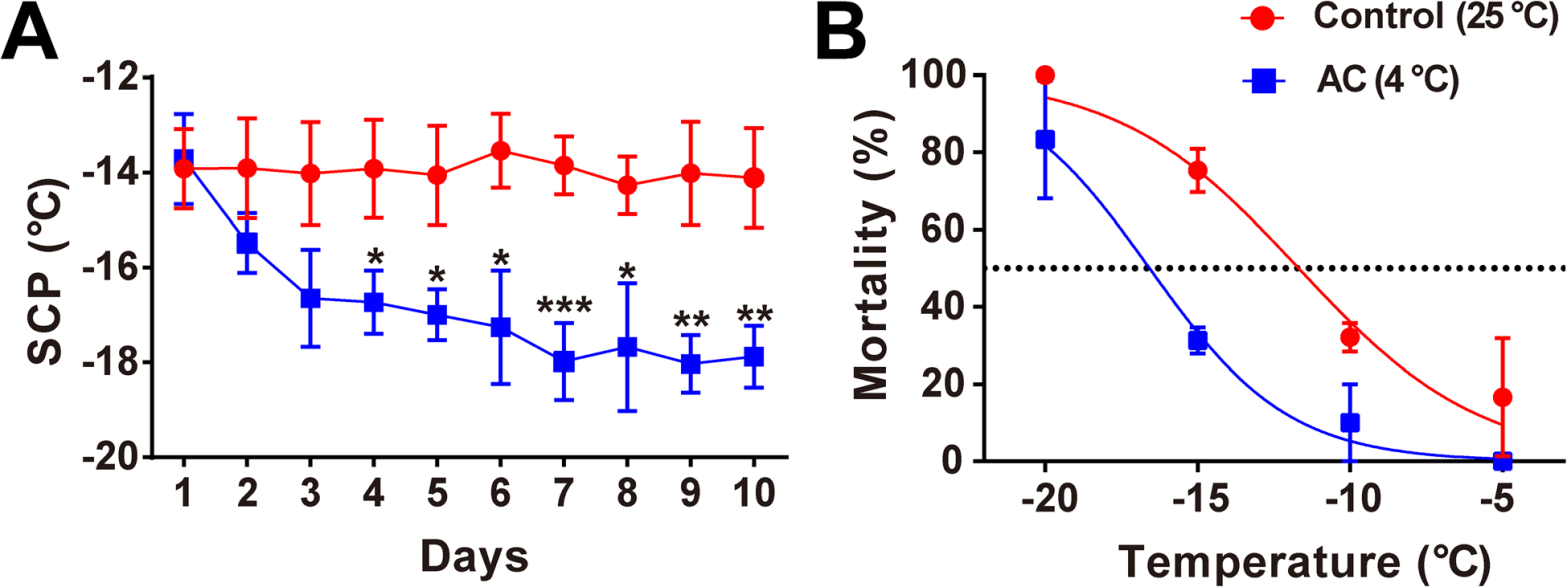
Cold acclimation enhances beetle cold hardiness. **a** Supercooling points (SCPs) of last-instar larval beetles were significantly lower after 4 days of acclimation at 4 °C than those of beetles held at 25 °C (*n* = 20 for each treatment per day). **b** Last-instar larval beetles acclimated to 4 °C for 10 days had significantly reduced mortality relative to those held at 25 °C (*n* = 10 for each treatment). Lethal temperature causing 50% mortality (LT_50_) was determined by probit regression. The data in **a** and **b** are shown as mean ± s.e.m. Two-tailed Student’s *t* test was used to test significant differences. *P < 0.05; **P < 0.01; ***P < 0.001. AC, low-temperature acclimation (4 °C); Control (25 °C). The last larval beetles used in **a** and **b** were collected at 20–22 Oct. 2016 and 20–25 Oct. 2019

To confirm whether low-temperature acclimation can enhance the cold hardiness of *Monochamus alternatus* beetles, we tested both SCPs and temperature causing 50% mortality (LT_50_). We made daily SCP measurements during 10 days’ acclimation to 4 °C, and measured mortality of larvae after 10 days of acclimation by exposing them to a series of low temperatures (− 20 °C, − 15 °C, − 10 °C, and − 5 °C) for 6 h, then determining LT_50_ for each treatment by probit regression. The SCPs of low-temperature-acclimated beetles dramatically decreased compared to those of control beetles after 4 days (Fig. 1a). Further, LT_50_ of acclimated beetles was 4.9 °C lower than that of controls (Fig. 1b; LT_50_: control: − 11.7 ± 0.5 °C, acclimated: − 16.6 ± 0.4 °C; extra sum-of squares *F* test: *F*_*1, 20*_ = 57.260, *P* < 0.001). Collectively, these results suggest that acclimation to 4 °C enhances cold hardiness in this beetle.

### miRNAs involved in the cold acclimation

**Fig. 2:**
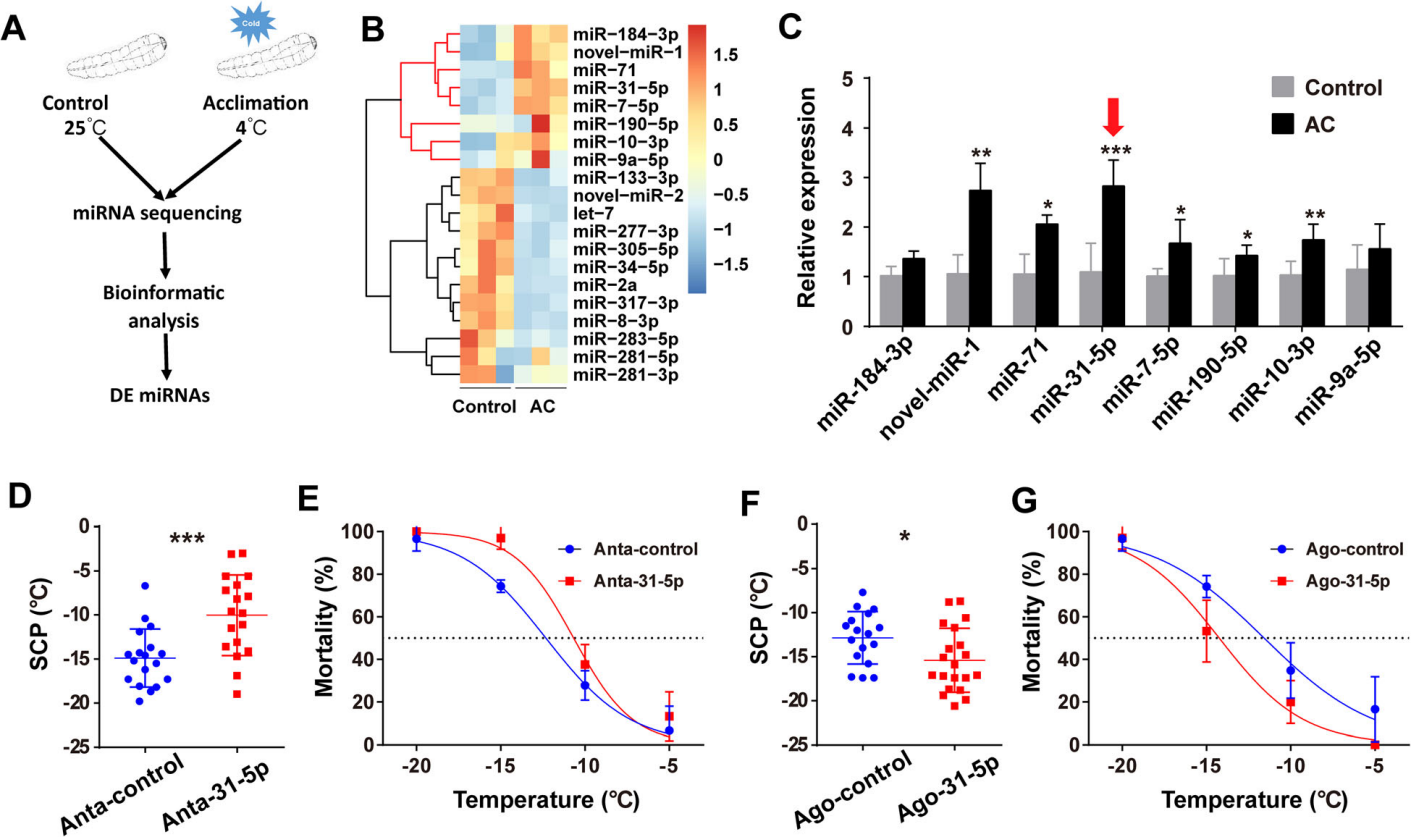
miRNAs involved in the beetle’s cold acclimation. **a** The procedure of screening for differentially expressed (DE) miRNAs as candidate regulators underlying cold acclimation. **b** Hierarchical clustering of the expression of the DE miRNAs for AC and control samples. The cluster in red represented the upregulated genes after AC. **c** The validation of relative expression of upregulated miRNAs after cold acclimation by qPCR (*n* = 6). miR-31-5p was selected as a candidate miRNA for further functional studies because it exhibits the greatest extent of upregulated expression as well as the highest abundance in larval beetles among the eight upregulated miRNAs. Two-tailed Student’s *t* test was used to test significant differences, *P < 0.05, **P < 0.01, ***P < 0.001. **d** Effect of antagomir-control (Anta-control, *n* = 18) and antagomir-31-5p (Anta-31-5p, *n* = 18) on supercooling points (SCPs) of larval beetles. Two-tailed Student’s *t* test was used to test significant differences, ***P < 0.001. **e** Effect of antagomir-control (Anta-control) and antagomir-31-5p (Anta-31-5p) on mortality of larval beetles (*n* = 9 for each treatment). Lethal temperature causing 50% mortality (LT_50_) was determined by probit regression. **f** Effect of agomir-control (Ago-control, *n* = 17) and agomir-31-5p (Ago-31-5p, *n* = 20) on SCPs of larval beetles. Two-tailed Student’s *t* test was used to test significant differences, *P < 0.05. **g** Effect of agomir-control (Ago-control) and agomir-31-5p (Ago-31-5p) mortality of larval beetles (*n* = 10 for each treatment). Lethal temperature causing 50% mortality (LT_50_) was determined by probit regression. AC, low-temperature acclimation (4 °C); Control (25 °C). The last larval beetles used in miRNA deep sequencing (**a**, **b**) were collected at 5 Oct. 2016, qPCR validation (**c**) and agomir injection (**f**) at 10 Oct. 2017, antagomir injection (**d**) at 15 Oct. 2017, and mortality experiments (**e**,** g**) at 20–25 Oct. 2019

We performed high-throughput sequencing of small RNAs of the acclimated and control last-instar beetle larvae (Fig. 2a). About 202–231 known miRNAs and 75–90 novel miRNAs were identified from 3 replicate control libraries and 3 replicate cold acclimated libraries, respectively (Additional file 1: Fig. S1A). Through comparative analysis, we found a total of 20 miRNAs to be differentially expressed (DE) between the acclimated and control beetles (Fig. 2b, Additional file 1: Fig. S1B, Additional file 2: Table S1; *P* value < 0.05 and fold change > 1.5). Their expression patterns were subsequently validated by quantitative PCR (qPCR) (Fig. 2c, Additional file 1: Fig. S2). Among the 8 markedly upregulated miRNAs expressed during low-temperature acclimation, miR-31-5p exhibited the greatest extent of expression upregulation (RPM in RNA-seq: 2.91-fold changes; qPCR: 2.58-fold changes) as well as the highest abundance (RPM in RNA-seq: 1369.30 ± 216.53 in control and 3984.73 ± 143.16 in the acclimated beetles) (Fig. 2c, Additional file 2: Table S2). This expression pattern indicated a potential role of miR-31-5p in cold acclimation of *M. alternatus* beetles.

We injected the chemically synthesized antagomir-31-5p or agomir-31-5p, which respectively silences and overexpresses endogenous miR-31-5p, into the hemolymph of the larval beetles, and subsequently measured their SCPs and LT_50_. We found antagomir-31-5p increased beetle SCP (Fig. 2d; *t* = − 3.656, *P* = 0.001) and LT_50_ (Fig. 2e; LT_50_: antagomir-control: − 12.3 ± 0.3 °C, antagomir-31-5p: − 10.7 ± 0.4 °C; extra sum-of squares *F* test: *F*_*1, 20*_ = 9.754, *P* = 0.005). Conversely, beetle’s SCP (Fig. 2f; *t* = 2.305, *P* = 0.027) and LT_50_ (Fig. 2g; LT_50_: agomir-control: − 11.6 ± 0.6 °C, agomir-31-5p: − 14.2 ± 0.5 °C; extra sum-of squares *F* test: *F*_*1, 20*_ = 11.930, *P* = 0.003) significantly decreased following injection of agomir-31-5p. These results indicated miR-31-5p is a key regulator of cold acclimation in *M. alternatus* beetles.

### Screening for gene targets of miR-31-5p

**Fig. 3:**
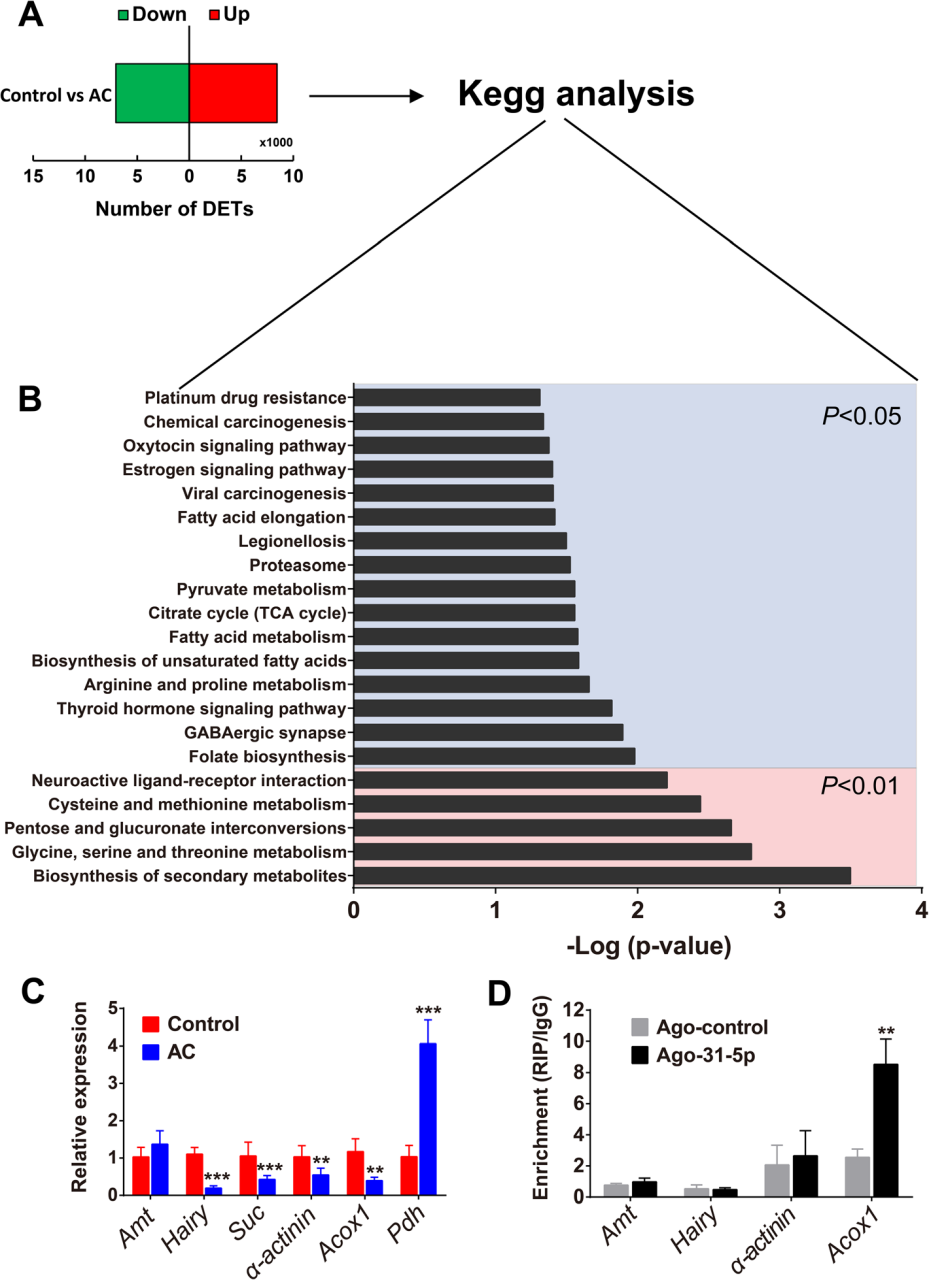
The screening of targets of miR-31-5p. **a** The number of up- and downregulated genes after low-temperature acclimation. **b** KEGG enrichment of the differentially expressed transcripts (DETs) after low-temperature acclimation. The genes in the clusters with *P* < 0.01 were used to predict the putative targets by miRanda and RNAhybrid algorithm. **c** mRNA level of candidate targets of miR-31-5p by qPCR (*n* = 6). Student’s *t* test was performed to test significant differences, **P < 0.01; ***P < 0.01. **d** RNA immunoprecipitation (RIP) was performed with an anti-AGO-1 antibody; normal rabbit IgG was used as a negative control. qPCR analysis was performed to quantify the relative expression level of the target genes from the immunoprecipitates treated with agomir-31-5p (Ago-31-5p) and agomir-control (Ago-control) (*n* = 6). Student’s *t* test was performed to test significant differences, **P < 0.01. Gene legends: *Amt*, aminomethyltransferase; *hairy*, hairy; *Suc*, succinate-CoA ligase; *α-actinin*, alpha actinin; *Acox1*, acyl-CoA oxidase; *Pdh*, proline dehydrogenase. AC, low-temperature acclimation (4 °C); Control (25 °C). The last larval beetles used in RNA-seq (**a**, **b**) were collected at 5 Oct. 2016, qPCR validation (**c**), and RIP (**d**) at 10 Oct. 2017

We conducted comparative transcriptome analysis between the acclimated and control last-instar beetle larvae by RNA-seq. In total, we found 15,486 differentially expressed transcripts (DETs) (fold change > 1.5, *P* value < 0.05), accounting for 25.8% of the total number of transcripts (59,971) in the reference transcriptome, of which 8439 were upregulated and 7047 were downregulated (Fig. 3a, Additional file 3: Dataset S1). KEGG analysis of these DETs showed 21 clusters were enriched (*P* < 0.05) (Fig. 3b, Additional file 3: Dataset S2). Among these clusters, five pathways including biosynthesis of secondary metabolites, glycine, serine and threonine metabolism, pentose and glucuronate interconversions, cysteine and methionine metabolism, and neuroactive ligand-receptor interaction were significantly enriched at the level of *P* < 0.01 (Fig. 3b). Among these pathways, 197 significantly enriched genes were designated as candidate genes (Additional file 3: Dataset S2). We employed the algorithms miRanda [28] and RNAhybrid [29] to predict the target genes of miR-31-5p and ultimately identified six putative target genes, namely *aminomethyltransferase* (*amt*), *hairy*, *succinate-CoA ligase* (*suc*), *alpha actinin* (*α-actinin*), *acyl-CoA oxidase 1* (*acox1*), and *proline dehydrogenase* (*pdh*).

RNA-seq and qPCR validation showed that *hairy*, *suc*, *α-actinin* and *acox1* were significantly downregulated (*hairy*: *t* = 5.119, *P* < 0.001; *suc*: *t* = 4.888, *P* < 0.001; *α-actinin*: *t* = 3.927, *P* = 0.002; *acox1*: *t* = 3.450, *P* = 0.005), whereas *pdh* was significantly upregulated (*t* = − 12.989, *P* < 0.001) and *amt* (*t* = − 1.818, *P* = 0.101) exhibited discrepancy between RNA-seq and qPCR after low-temperature acclimation (Fig. 3c). Given that the miRNA commonly negatively regulates target genes in animals [30], we selected the four downregulated genes that exhibited an opposite expression pattern with miR-31-5p for further validation (Fig. 3c).

Argonaute (Ago) proteins are key RNA-binding proteins in RNA-induced silencing complexes (RISCs) when miRNAs regulate the mRNAs at the post-transcriptional level [31]. Thus, we assessed the interaction between Ago and each of the four genes in vivo by RNA immunoprecipitation (RIP) assays using beetles injected with agomir-31-5p or agomir-control. Only *acox1* was enriched in Ago1-immunoprecipitated compounds from agomir-31-5p-treated beetles compared with agomir-control-treated beetles (Fig. 3d; *t* = − 3.516, *P* = 0.009). Therefore, *acox1* is most likely regulated by miR-31-5p.

### miR-31-5p negatively targets the CDS of *acox1*

**Fig. 4:**
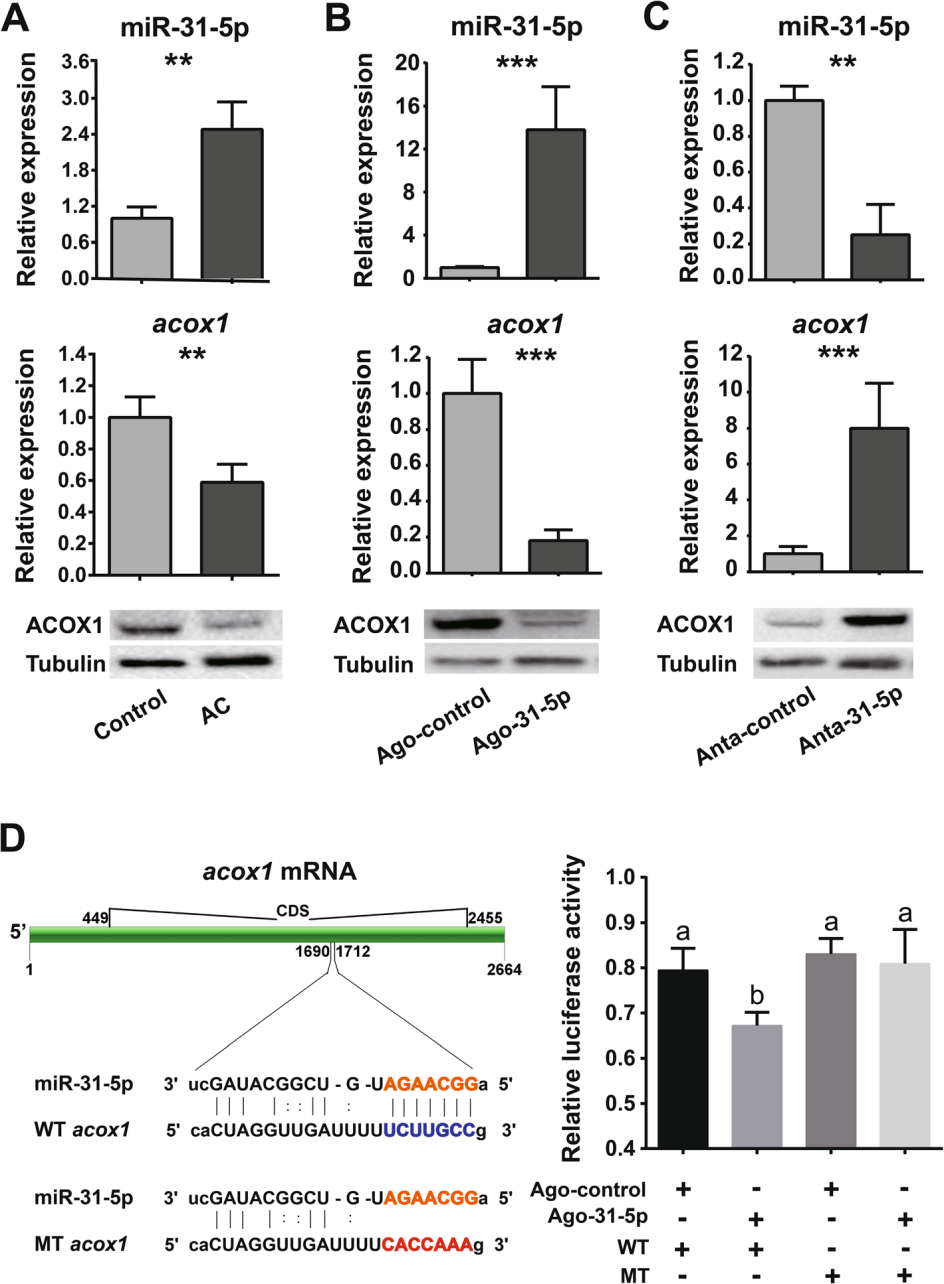
miR-31-5p directly targets *acox1*. **a** The relative expression of miR-31-5p, *acox1* mRNA, and protein after low-temperature acclimation by qPCR (*n* = 6) and Western blot (*n* = 3). The bar graphs show mean ± s.e.m. (**, *P* < 0.01; ***, *P* < 0.001; Student’s *t* test). **b** The relative expression of miR-31-5p, *acox1* mRNA, and protein after injecting agomir-31-5p (Ago-31-5p) and agomir-control (Ago-control) by qPCR (*n* = 6) and Western blot (*n* = 3). **c** The relative expression of miR-31-5p, *acox1* mRNA, and protein after injecting antagomir-31-5p (Anta-31-5p) and antagomir-control (Anta-control) by qPCR (*n* = 6) and Western blot (*n* = 3). **d** Luciferase reporter assays in S2 cells co-transfected with agomir-31-5p or agomir-control and psiCHECK2 vectors containing wild (WT) or mutant (MT) sequences of predicted target sites. The bars are means ± s.e.m. and differ significantly from each other with different letters (*n* = 8, one-way ANOVA with Tukey’s HSD multiple comparison, *P* < 0.05). AC, low-temperature acclimation (4 °C); Control (25 °C). The last larval beetles used in **a**, **c** were collected at 10–15 Nov. 2017, and beetles in **b** and **d** were collected at 5 Oct. 2017

We further tested the expression patterns of miR-31-5p and *acox1* mRNA by qPCR, and the protein expression level by Western blot following low-temperature acclimation and injection of agomir-31-5p and antagomir-31-5p, respectively. Low-temperature acclimation caused miR-31-5p upregulation but downregulation of *acox1* mRNA and its protein (Fig. 4a; miR-31-5p: *t* = − 4.162, *P* < 0.003; *acox1* mRNA: *t* = 5.020, *P* = 0.006). After injection of agomir-31-5p, the expression of miR-31-5p increased while the expression of *acox1* mRNA and protein decreased (Fig. 4b; miR-31-5p: *t* = − 10.365, *P* < 0.001; *acox1* mRNA: *t* = 12.385, *P* < 0.001). In contrast, the expression of miR-31-5p, *acox1* mRNA, and its protein in antagomir-31-5p treatments exhibited the opposite expression pattern with miR-31-5p downregulation and upregulation of *acox1* mRNA and protein (Fig. 4c; miR-31-5p: *t* = 4.352, *P* = 0.005; *acox1* mRNA: *t* = − 8.911, *P* < 0.001). These results confirmed the negative relationship between miR-31-5p and *acox1* mRNA and protein.

Furthermore, we predicted the target sites of mal-miR-31-5p in *acox1* orthologs from *M. alternatus* and other model animals including *Homo sapiens*, *Mus musculus*, *Drosophila melanogaster*, and *Caenorhabditis elegans* (Additional file 1: Fig. S3A). The results showed that most (except for Mal-*acox1b*, Cel-*acox1.2*, and Cel-*acox1.3*) of the selected genes harbored the target site of mal-miR-31-5p in either coding sequence (CDS) regions (*M. alternatus*, *D. melanogaster*, and *C. elegans*) or 3′ UTR regions (*H. sapiens* and *M. musculus*) (Additional file 1: Fig. S3B). Though the relative position of these target sites in CDS was variant (Additional file 1: Fig. S3C), it is at least suggested the miR-31-5p-*acox1* axis may be conserved across the Animal Kingdom.

The presence of a putative binding site in the coding sequences suggests a possible direct regulation between miR-31-5p and *acox1* mRNA in *M. alternatus* (Fig. 4d, Additional file 1: Fig. S3B). We tested this hypothesis by performing luciferase assays in *Drosophila* S2 cells. Luciferase activity from the constructs containing the predicted target site (wild type, WT) from coding sequences (CDS) of *acox1* decreased significantly after injection of agomir-31-5p compared to those injected with agomir-control. Furthermore, luciferase activity of the constructs harboring a seed sequence-mutated binding site had no difference after injection of agomir-31-5p compared to those injected with agomir-control (Fig. 4d; one-way ANOVA with Tukey’s HSD: *F*_*3, 23*_ = 6.555, *P* = 0.013). Collectively, these results suggested miR-31-5p directly targeted *acox1* in a negative manner.

### miR-31-5p-*acox1* promotes asc-C9 accumulation and cold acclimation

**Fig. 5:**
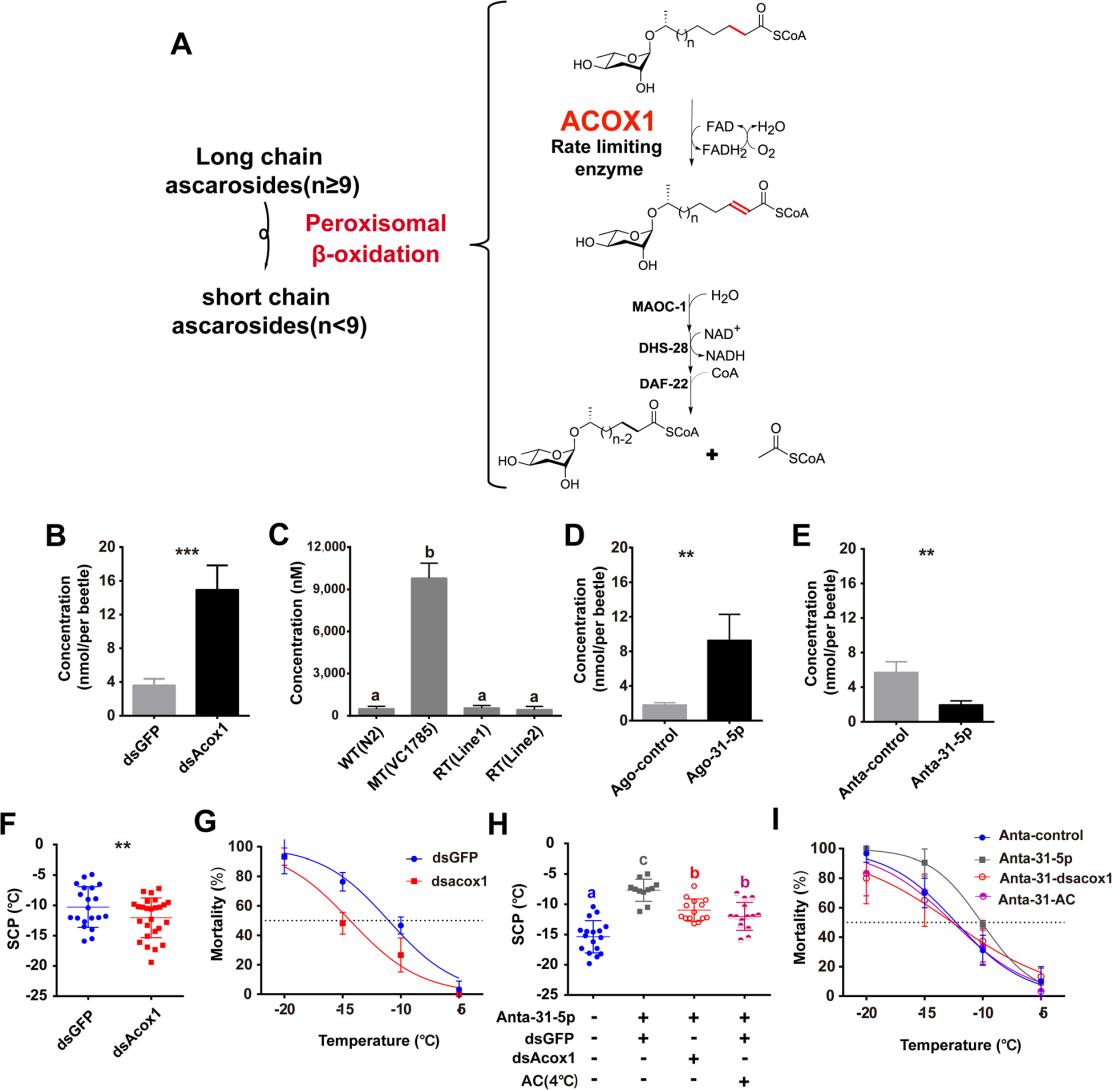
miR-31-5p-*acox1* promotes beetle’s asc-C9 accumulation and cold acclimation. **a** ACOX1 is the rate-limiting enzyme in peroxisomal β-oxidation cycles which function in ascaroside side chain shortening in *C. elegans*. Modification from Zhang et al. [32]. **b** The amount of asc-C9 after RNAi by injecting dsAcox1 and dsGFP (Control) in larval beetles (*n* = 8). ***, *P* < 0.001; Student’s *t* test. **c** Comparison of the concentration of asc-C9 secreted by the wild type (WT, N2 strain), *acox1* loss of function mutant (MT, VC1785 strain), and rescue type (RT, *acox1* (ok2257):: Cel-p:: Mal*-acox1*, line 1 and line 2) of *C. elegans* using HPLC-MS/MS. The rescue types were constructed from the *acox1* mutant *C. elegans* with heterologous expression of Mal-*acox1* through transforming the pPD95_77 plasmid cloned with the promoter of Cel-*acox1* (Cel-p) and CDS of Mal-*acox1* into *C. elegans* mutant. The bars are means ± s.e.m. and differ significantly from each other with different letters (*n* = 8, one-way ANOVA with Tukey’s HSD multiple comparison, *P* < 0.05). **d**, **e** The amount of asc-C9 after injecting the agomir-31-5p (Ago-31-5p) (**d**) and antagomir-31-5p (Anta-31-5p) (**e**) in larval beetles (*n* = 8). **, *P* < 0.01; Student’s *t* test. **f** The supercooling points (SCPs) of larval beetles after RNAi by injecting dsAcox1 (*n* = 26) and dsGFP (Control) (*n* = 20). **, *P* < 0.01; Student’s *t* test. **g** Probit regression between mortality rate and temperature after injecting dsGFP and dsAcox1 (*n* = 10 for each treatment). **h** The supercooling points (SCPs) of wild type larval beetles (25 °C) (*n* = 17), antagomir-31-5p injected beetles (*n* = 11), and the rescue beetles by injecting dsAcox1 (*n* = 14) or low-temperature acclimation (*n* = 14). The bars with different letters differ significantly from each other (one-way ANOVA with Tukey’s HSD multiple comparison, *P* < 0.05). **i** Probit regression between mortality rate and temperature for antagomir-control-injected beetles, antagomir-31-5p (Anta-31-5p)-injected beetles, and the rescue beetles by injecting dsAcox1 (Anta-31-dsacox1) or low-temperature acclimation (Anta-31-AC) (*n* = 12 for each treatment). Control (25 °C); AC, low-temperature acclimation (4 °C). The last larval beetles used in **b** were collected at 15 Oct. 2017, **d**, **f** at 5 Oct. 2017, **e**, **h** at 6 Nov. 2017, and **g**, **i** at 20–25 Oct. 2019

ACOX-1.1 in *C. elegans* is a homolog of ACOX1, the rate-limiting enzyme in the peroxisomal β-oxidation cycle, and is well known to desaturate the CoA thioesters of (ω-1)-ascarosides with medium- and long-fatty acid side chains (e.g., asc-C9) in nematodes [32] (Fig. 5a). Asc-C9 can also be produced by *M. alternatus* [23], and its amount significantly increased after the larvae were subjected to both laboratory cold acclimation (Additional file 1: Fig. S4A) and field cooling over time in autumn (Additional file 1: Fig. S4B). Thus, we hypothesized that miR-31-5p-ACOX1 may be linked to the accumulation of asc-C9 in beetles. To test this hypothesis, we first determined the function of ACOX1 in *M. alternatus* (Mal-ACOX1) with regard to asc-C9 accumulation by RNA interference. Asc-C9 accumulated much higher in beetles injected with double-strand *acox1* (dsAcox1) compared with those injected with double-strand green florescent protein (dsGFP, control) (Fig. 5b; *t* = − 10.736, *P* < 0.001). To provide further evidence, we constructed two rescue types (RT), line 1 and line 2, through heterologous expression of Mal-*acox1* in the mutant of *C. elegans*. Afterwards, we compared the amount of asc-C9 secreted by nematodes of wild type (WT), *acox1* loss of function mutant (MT) and rescue types (RTs) line 1 and line 2 in liquid cultures. MT nematodes secreted much more asc-C9 (> 10-fold) than WT (Fig. 5c). After the heterologous expression of Mal-*acox1*, the asc-C9 secreted by RTs line 1 and line 2 decreased sharply compared with that of MT, but were equal to that of WT (Fig. 5c; one-way ANOVA with Turkey’s HSD: *F*_*3, 17*_ = 69.316, *P* < 0.001). RNAi and heterologous expression jointly suggested that *acox1* negatively regulated asc-C9 accumulation in beetles. With respect to miR-31-5p, we compared the amount of asc-C9 from beetles injected with agomir-31-5p (agomir-control) and antagomir-31-5p (antagomir-control), respectively. The asc-C9 significantly increased after injections of agomir-31-5p (Fig. 5d; *t* = − 4.766, *P* = 0.008) and conversely decreased after treatment with antagomir-31-5p (Fig. 5e; *t* = 3.212, *P* = 0.009), suggesting the regulation of asc-C9 by miR-31-5p.

We further tested the effect of *acox1* on the beetle’s SCPs and LT_50_ by RNAi and a series of rescue experiments to confirm the functions of miR-31-5p-*acox1* axis. The SCPs (Fig. 5f; *t* = 2.702, *P* = 0.01) and LT_50_ (Fig. 5g; dsGFP: − 11.1 ± 0.5 °C, dsAcox1: − 14.4 ± 0.5 °C; extra sum-of squares *F* test: *F*_*1, 20*_ = 19.250, *P* < 0.001) of beetles injected with dsAcox1 were significantly lower than that of beetles injected with dsGFP, indicating enhancement of cold hardiness. The rescue experiments showed that the SCPs of beetles injected with antagomir-31-5p significantly increased compared to controls. After RNAi was conducted on *acox1* or low-temperature acclimation was performed in antagomir-31-5p-injected beetles, their SCPs evidently declined in both experiments, though the SCPs were still higher compared to that of controls (Fig. 5h; one-way ANOVA with Turkey’s HSD: *F*_*3, 53*_ = 15.233, *P* < 0.001). LT_50_ of beetles injected with antagomir-31-5p was also significantly higher than that of beetles in control, RNAi of *acox1* and cold acclimation after injecting antagomir-31-5p (Fig. 5i; extra sum-of squares *F* test: *F*_*3, 40*_ = 5.501, *P* = 0.003). These results suggest miR-31-5p-*acox1* axis mediated the beetle’s cold hardiness. Taken together, miR-31-5p-*acox1* axis simultaneously promotes the accumulation of asc-C9 and the cold hardiness of *M. alternatus* beetles, implying a positive correlation between asc-C9 and cold acclimation.

### Asc-C9 promotes the beetle’s cold acclimation

**Fig. 6:**
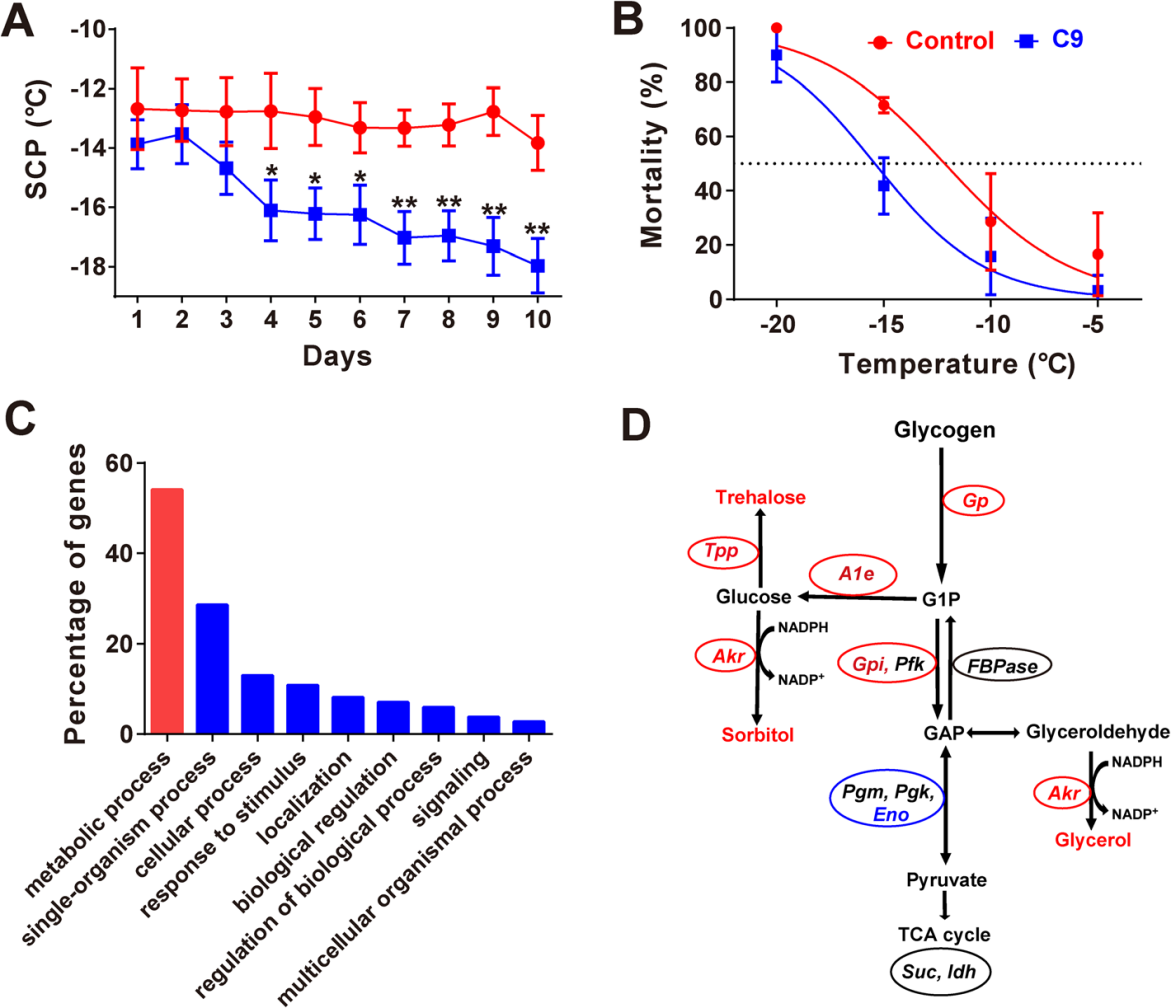
Asc-C9 acts as a cold signal to promote beetle’s cold acclimation. **a** The effect of asc-C9 on the supercooling points (SCPs) of the larval beetles (*n* = 20). **b** Probit regression between mortality rate and temperature of asc-C9-fed and control beetles (*n* = 10 for each treatment). **c** Distribution of Gene Ontology (GO) terms with > 1% of annotated genes in the category of biological process enriched from the DETs between asc-C9-fed and control beetles. **d** Simplified schematic diagram of the synthesis and metabolism of these three cryoprotectants modified from Barth et al. [33]. The genes in red are significantly upregulated and those in blue are significantly downregulated, and those in black have no changes in expression after treatment of asc-C9. Genes legends: *Tpp*, trehalose-phosphate phosphatase; *Gp*, glycogen phosphorylase; *A1e*, aldose-1-epimerase; *Akr*, aldo-keto reductase; *Gpi*, glucose-6-phosphate isomerase; *Pfk*, 6-phospho-fructokinase; *Suc*, succinyl-CoA ligase; *Eno*, enolase; *Idh*, isocitrate dehydrogenase; *FBPase*, fructose-1,6-bisphosphatase; *Pgm*, phosphoglycerate mutase. G1P, glucose-1-phosphate; GAP, glyceraldehyde-3-phosphate. The data were shown as mean ± s.e.m. Two-tailed Student’s *t* test was used to test significant differences. *P < 0.05; **P < 0.01; ***P < 0.001. C9, asc-C9-fed beetles (4 °C); Control beetles (25 °C). The last larval beetles in **a** and **b** were collected at 6–8 Oct. 2018 and 20–25 Oct. 2019, respectively

We tested the direct effect of ascarosides on the beetle’s cold hardiness by feeding larval beetles with synthetic asc-C9, asc-△C6, or water (control) grown on artificial diets and measured their SCPs daily and LT_50_ of beetles on the 10th day after treatment. The SCPs of asc-C9-fed beetles began to markedly decrease from the 4th day after treatment (Fig. 6a). Moreover, LT_50_ of asc-C9-fed beetles was 3.2 °C lower than that of controls (Fig. 6b; LT_50_: control: − 12.2 ± 0.7 °C, asc-C9-fed: − 15.4 ± 0.5 °C; extra sum-of squares *F* test: *F*_*1, 20*_ = 22.730, *P* < 0.001), while SCPs and LT_50_ of asc-△C6-fed beetles had no differences compared with controls (Fig. S5A, B). These results suggest the potential roles of this asc-C9 in the beetle’s cold hardiness.

To further characterize specific gene expression patterns after asc-C9 treatment, we conducted comparative transcriptome analysis between the asc-C9-fed and control last-instar beetle larvae by RNA-seq. In total, we found 1601 differentially expressed transcripts (DETs) (fold change > 1.5, *P* value < 0.05), of which 446 were upregulated and 1155 were downregulated after asc-C9 treatment (Additional file 1: Fig. S6A, Additional file 3: Dataset S3). Gene Ontology (GO) analysis of these DETs showed “metabolic process” with 100 genes accounting for 54.05% of the annotated DETs, was the most frequent GO term in the category of biological process (Fig. 6c, Additional file 3: Dataset S4), indicating the dramatic effect of asc-C9 on beetle’s metabolism. Among these enriched 100 genes in metabolic process, 78 were downregulated and 22 were upregulated (Additional file 3: Dataset S4). In addition, most genes, which were classified into the 12 significantly enriched sub-GO terms (*P* < 0.05), were downregulated (percentage of downregulated genes in each sub-term ranges from 50 to 100%) (Additional file 1: Fig. S6B; Additional file 3: Dataset S5), suggesting a strong metabolic depression.

Expression analysis of cryoprotectant-associated genes by both RNA-seq and qPCR showed some genes encoding the enzymes for cryoprotectant synthesis, including trehalose-phosphate phosphatase (*Tpp*), glycogen phosphorylase (*Gp*), aldose-1-epimerase (*A1e*), aldo-keto reductase (*Akr*), and glucose-6-phosphate isomerase (*Gpi*), were significantly upregulated (Additional file 1: Fig. S6C, D). Conversely, enolase (*Eno*), a gene encoding the enzymes for tricarboxylic acid cycle (TAC cycle), was significantly downregulated. This expression pattern suggested accumulation of cryoprotectants such as glycol, trehalose, and sorbitol (Fig. 6d).

## Discussion

**Fig. 7:**
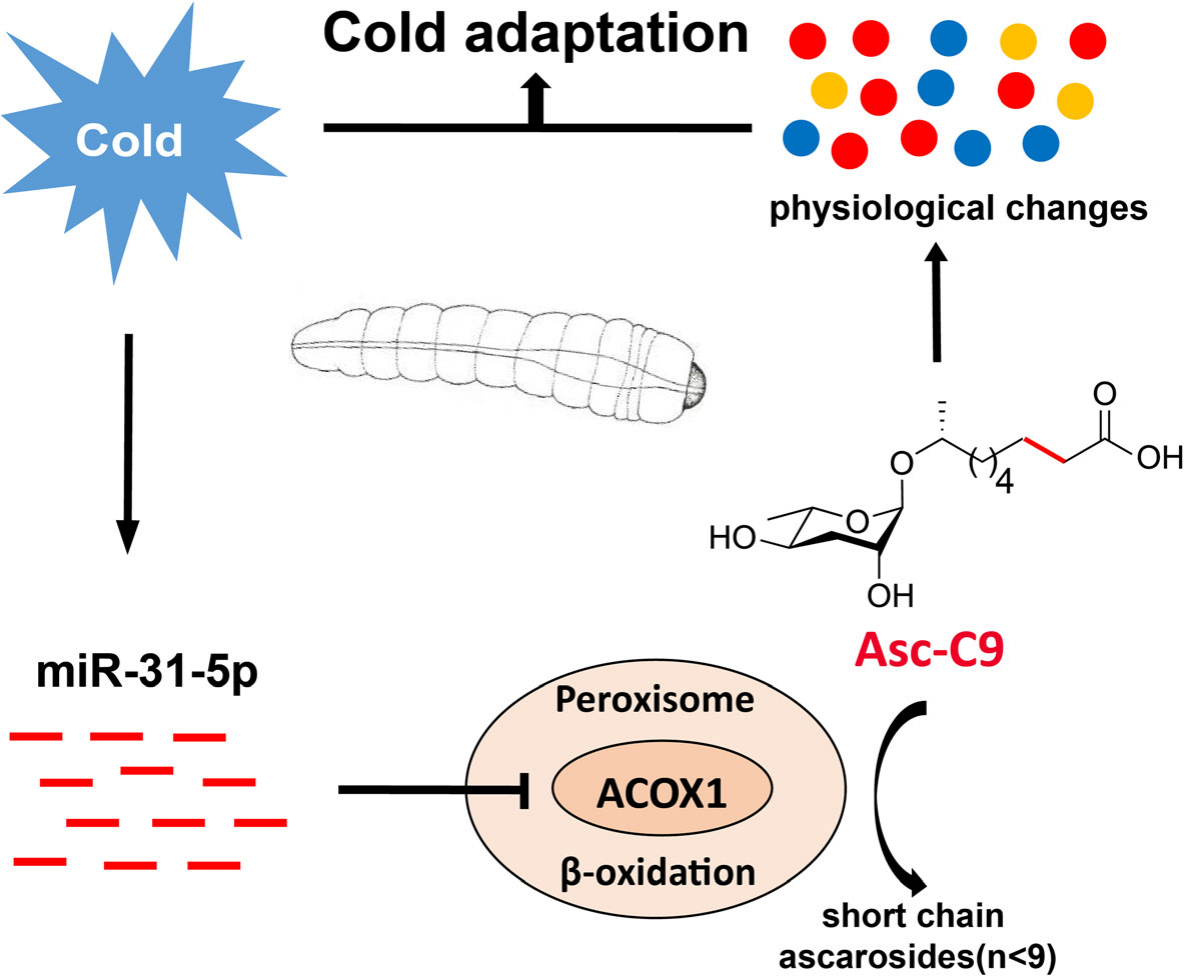
Schematic illustration of cold acclimation regulated by miR-31-5p via asc-C9 in *Monochamus alternatus*

In this study, we found a novel function of thermal-dependent ascarosides that can serve as a secondary cold signal to facilitate cold acclimation in *M. alternatus*. Mechanistically, upon sensing low temperature, asc-C9 production was promoted by miR-31-5p through negatively targeting a rate-limiting enzyme, ACOX1, in the peroxisomal β-oxidation cycle for shortening of the side chains of the ascarosides. Asc-C9 appears to further regulate cold physiology by altering gene expression linked to metabolic depression and cryoprotectant accumulation, ultimately enhance cold hardiness in this beetle (Fig. 7). These results reveal a novel molecular mechanism by which a miRNA-mediated small signaling molecule promotes cold acclimation in an insect.

Since the first report of the involvement of ascarosides in dauer formation in *C. elegans* [34], ascarosides have been shown to be multi-functional signaling molecules regulating male attraction, hermaphrodite repulsion, olfactory plasticity, aggregation, and lipid metabolism in nematodes [35, 36]. In addition, ascarosides were also shown to coordinate the life cycles between the pinewood nematode and its vector beetle, thus maintaining the invasive symbiosis and promoting the dispersal of pinewood nematodes [23]. Here we further demonstrated the first reported function of these small molecules in cold acclimation in *M. alternatus*.

Insect cold hardiness is often achieved by physiological changes [5, 6], which are mostly linked to specific gene expression patterns [33, 37]. Here, we characterized a gene expression pattern of metabolic depression and accumulation of cryoprotectants after asc-C9 treatment. Metabolic depression is essential for conserving energy stores and thus could enhance resistance to cold stress [9, 14, 16]. Low molecular weight polyols and sugars including glycerol, trehalose, and sorbitol have been reported as cryoprotective agents in many insects [4, 8]. The functions of these compounds include both colligative depression of supercooling point (SCP) and non-colligative stabilization of membranes and proteins [6]. The gene expression patterns of metabolic depression and cryoprotectant accumulation documented here thus are typical for cold hardy insects, and further supported the important role of ascaroside in enhancement of the beetle’s cold hardiness. Since low winter temperature is a limiting factor to the distribution and potential dispersal areas in this beetle [38], the enhanced cold tolerance allowing higher survival of *M. alternatus* in winter and might have shaped its wide distribution range that occupies a large latitudinal span across Indomalayan and Palearctic realms, which has dramatic implications for the future spread of PWN and pine wilt disease [39].

It is well known that chemical signals, especially the glandular-produced hormones like juvenile hormone (JH) and ecdysone, are largely involved in cold stress responses in insects [14]. Additionally, several metabolites such as soluble sugars, the tetrapyrrole intermediate Mg-protoporphrin (Mg-ProtoIX), and ROS have also been suggested to be chemical signals involved in cold adaptation in plants [40–43]. However, metabolite-derived stress signals are almost completely unknown in insects. Here, we provide experimental lines of evidences that an ascaroside is likely to be a metabolic signal for cold acclimation, and this is supported by the following two reasons: First, asc-C9, a small molecule derived from metabolites, increased in abundance in response to decreased temperatures, illustrating the feature that metabolic signals have thermal-dependent plasticity; second, feeding with trace synthetic asc-C9 can significantly altered gene expression patterns of metabolic depression and cryoprotectant accumulation in our study, and retards the development of the beetle by reducing ecdysone, slowing it down for pupation [23]. Lastly, many analogs of ascarosides with different side chain lengths have been identified and quantified in insects from several orders (unpublished data from the authors), implying these small signaling molecule might widely participate in cold adaptation in other insects.

miRNA-mediated stress adaptation has been documented in several studies [20]. Here, we obtained the first miRNA library of *M. alternatus* beetles and described a special miRNA-mediated cold adaptation response where miR-31-5p regulates insect cold acclimation via regulation of the production of a cold signal (ascaroside) rather than directly targeting the stress responsive genes or their transcription factors [44]. Specifically, upregulated miR-31-5p under cold stress promoted the accumulation of asc-C9 by negatively targeting *acox1*, the first and rate-limiting enzyme gene in peroxisomal β-oxidation, resulting in enhanced cold hardiness by metabolic modification. Targeting a signal makes the miRNA more effectively promote cold acclimation compared to that of targeting a single responsive gene, as the signal can synchronously coordinate a series of pathways underlying this process [45].

The antagonistic relationship of miR-31-5p and *acox*1 was also recently confirmed in humans [46], but the binding sites of miR-31-5p were located in different regions: the 3′ untranslated region (3′ UTR) in humans [46], whereas it is found in the coding sequences (CDS) in *M. alternatus*, suggesting that miR-31-5p-*acox1* axis may be conserved across the Animal Kingdom. The family of Acyl-CoA oxidases (ACOX), the first and rate-limiting enzymes of β-oxidation in peroxisome, exhibits substrate specificity [32, 47, 48]. In *C. elegans*, there are at least seven ACOXs encoded by its genome (cel-ACOX1.1, cel-ACOX1.2, cel-ACOX1.3, cel-ACOX1.4, cel-ACOX1.5, cel-ACOX1.6 and cel-ACOX3), in which cel-ACOX1.1 homodimers are specific for the desaturation of ascarosides with nine and longer carbon side chains, cel-ACOX1.1/cel-ACOX3 heterodimers oxidize ascarosides with seven or fewer carbons on the chain, ACOX-1.4 processes ascarosides with 9- and 11-carbon side chains, and ACOX-3 processes ascarosides with 13- and 15-carbon side chains, while cel-ACOX1.2 homodimer controls the production of those with ω-side chains with less than five carbons [32, 49]. In the present study, we found three transcripts encoding ACOXs (ACOX1, ACOX1b and ACOX3) from the transcriptome of *M. alternatus*. Among them, only *acox1* gene harbors the target sequence of miR-31-5p. Functional analysis by RNAi, injection of agomir-31-5p, and heterologous expression of Mal-*acox*1 in mutant *C. elegans* (VC1785) suggested that asc-C9 is one of the specific substrates of ACOX1 in insects, suggesting functional conservation of ACOXs in the production of small molecule ascarosides.

## Conclusions

Overall, we addressed an important and under-explored aspect of insect cold acclimation that a miRNA-mediated ascaroside serves as a metabolic cold signal and promotes insect cold hardiness via shaping the expression of genes involved in metabolic depression and accumulation of cryoprotectants. Effective cold adaptation enables both the vector insect and pinewood nematode to have a wide distribution range and leads to further range expansion of pine wilt disease, which may be exacerbated by climate change. These results thus can also inspire the development of miRNA or ascaroside-based pest management for this important quarantine pest. Furthermore, the possible involvement of ascarosides in cold adaptation of other insects is worthy of further exploration.

## Methods

### Beetle collection and rearing

Final (5th) instar larval *Monochamus alternatus* were collected from host trees of *Pinus massoniana*, in Fuyang, Zhejiang province, in late autumn (i.e., 1 October–15 November) from 2016 to 2018, when the larval beetles begin to face lower temperatures and initiate cold acclimation, but have not yet completed cold acclimation nor entered into diapause. Additionally, the amount of asc-C9 of these larval beetles peaks during this period [23]. Thus, this instar is the ideal stage for the experimental cold acclimation and ascaroside analysis. Beetles were brought to the laboratory and reared on artificial diet (200 g of sawdust, 15 g of agar, and 550 ml of distilled water) in a 10-ml tube at a 12:12 h light:dark (L:D) cycle at 25 °C placed in a climate chamber. Fresh diet was provided every week. These beetles were used for all of the subsequent experiments unless otherwise noted.

A previous study has shown that *M. alternatus* has seasonal variation in super cooling points [24]. Meanwhile, our study proved the amount of ascaroside (asc-C9) produced by the last-instar beetle larvae significantly changes with cold acclimation in the laboratory (artificial cold acclimation) and at different time points depending on the collection date during late autumn (i.e., natural cold acclimation) in field (Additional file 1: Fig. S4A, B). To eliminate the effect of these variations, we standardized the beetle larvae used in each experiment by only using beetles that were field collected at the same time.

### Experimental acclimation, measurement of SCPs, and mortality rate

To examine the effect of low temperature on the beetle’s cold hardiness, 200 final (5th instar) larval beetles with weights ranging from 300 to 500 mg were individually placed in a climate chamber at 4 °C for experimental cold acclimation [23, 24], with 200 individuals at 25 °C for non-acclimation as control. Except for the temperature, the other parameters were the same as their rearing conditions.

SCP is the lowest temperature at which an abrupt rebound on the thermal curve occurs due to the release of the latent heat of ice crystallization in an insect [24]. Twenty individuals were sampled for their super cooling point (SCP) measurement from both cold acclimation experiments and asc-C9-feeding experiments every day for 10 days. SCPs were measured using an insect SCP tester (SUN-V, Pengcheng Electronics, Beijing, China) with a refrigerator (BC/BD 326C, Haier, China). Specifically, the larvae was placed in a 1.5-ml tube and attached to a thermocouple of the tester, then fixed and enclosed by a piece of cotton [50]. The thermocouple with the larva was placed into a freezing chamber that was cooled gradually at a rate of ca. 1 °C/min during measurements. The larva’s body temperature decreased in a nonlinear way, the SCP being represented on the recorder by a sudden spike in the temperature of the thermocouple. Data were automatically recorded using customized software (Pengcheng Electronics, Beijing). Twenty replicates were used for each treatment.

Mortality experiments were conducted as previously described [24, 50] with modification. Specifically, larval beetles from each treatment were exposed to one of four different cold constant temperatures (− 20 °C, − 15 °C, − 10 °C, and − 5 °C) in refrigerators for 6 h, respectively. After that, all larvae were placed in a climate chamber (25 °C, light:dark = 12:12 h) to recover for 1 day. Death was assessed by the absence of mandibular or body movement when larvae were stimulated with a needle. Finally, the lethal temperature causing 50% mortality (LT_50_) for each treatment was determined by probit regression. Nine to twelve individuals were tested in each treatment. Each treatment had three replicates.

### High-throughput sequencing of small RNAs and RNA-seq

To examine the miRNAs and mRNAs involved in the cold acclimation and ascaroside feeding experiments, the acclimated (4 °C)/control (25 °C) and asc-C9-fed/control larval beetles (weight: 400 ± 20 mg) were respectively collected after 10 days of treatment for high-throughput sequencing of small RNAs and RNA-seq, respectively. Three replicates were used for each treatment.

Total RNA from the whole body was extracted by TRIzol Reagent (Invitrogen, USA). Small RNA and mRNA libraries were constructed using a TruSeq small RNA sample preparation kit (Illumina, USA) and RNA Library Prep Kit (Illumina, USA), respectively. The libraries were sequenced on the platform of Illumina HiSeq2500 at Bionova Biotech Company (Beijing). Raw data of the libraries were deposited in the NCBI Sequence Read Archive (accession number: PRJNA522884).

After quality control, bioinformatics analyses were performed. For miRNAs, the remaining reads were clustered based on sequence similarity and dominant reads were analyzed by bowtie (version 2.3.4) [51], against Genbank and Rfam to discard rRNA, scRNA, snoRNA, snRNA, and tRNA. Subsequently, known miRNAs were identified by mapping to the miRNA sequences from insects in miRBase 22.1, and novel miRNAs were predicted using miRDeep2 (version 2.0.1.2) [52]. Differential expression analysis of miRNAs was performed using the DEGseq method [53], and differentially expressed miRNAs were finally selected with fold change > 1.5 and *P* < 0.05. With respect to RNA-seq, the filtered data were assembled by Trinity (version 2.6.5) [54], to obtain reference transcripts due to the reference transcriptome (PRJNA374773). The differentially expressed transcripts (DETs) were analyzed using Cuffdiff software (version 7) [55], and differentially expressed mRNAs were finally selected with fold change > 1.5 and *P* < 0.05. GO and KEGG enrichments were performed with cut-off *P* values of 0.01 and 0.05, respectively (Additional file 3: Dataset S6).

### Predicted targets of miR-31-5p

miRanda and RNAhybrid were employed to predict the targets of miR-31-5p in the significantly enriched genes (*P* < 0.01; Additional file 3: Dataset S2) by KEGG analysis of cold related DETs (Additional file 3: Dataset S1). An alignment score greater than 154 was used in miRanda [28]. The parameters of RNAhybrid were set as 1 hit per target, free energy threshold was − 25 kcal mol^− 1^, helix constraint was 2 to 7, max bulge loop length was 2, max internal loop length was 6, and no G:U in seed is included [56]. The overlapped genes predicted through both methods were selected.

### qPCR for mRNA and miRNA

Total RNA was isolated from the whole body by TRIzol (Invitrogen, USA). Two micrograms of total RNA from each sample was reverse-transcribed using a miRcute miRNA First-Strand cDNA Synthesis Kit (Tiangen, China) and the FastQuant RT Kit with gDNase (Tiangen) according to the manufacturers’ instructions for miRNA and mRNA, respectively. The relative expression of miRNAs and mRNAs was respectively quantified by the miRcute miRNA qPCR Detection Kit (Tiangen) and a Real Master Mix Kit (SYBR Green) (Tiangen) with an MX3000P Thermal cycler (Stratagene, USA) instrument. The programs of thermal cycling were set following the manufacturer’s instructions for miRNA: 95 °C for 15 min, 40 cycles at 94 °C for 20 s, 60 °C for 34 s; mRNA: 95 °C for 1.5 min, 40 cycles at 94 °C for 15 s, 55 °C for 20 s, 68 °C for 60 s. To check for the specificity, melting curves were analyzed for each data point. The primers used here are included in Additional file 3: Dataset S7. U6 snRNA and β-actin were respectively selected as reference genes from 5 miRNAs and 1 snRNA, and 3 housekeeping genes examined following expression stability analysis using the Excel 2007 add-in NormFinder software (version 0.953) [57]. The expression values were calculated using the 2^−ΔΔCT^ method [58]. Six biological replicates and three technical replicates were performed for statistical analysis.

### Western blot

Polyclonal antibody against ACOX1 was developed through injection of the KLH protein-coupled polypeptide “CTQKSPLNAEQVNKS” into rabbits, which is specific for ACOX1, but lacks in ACOX1b and ACOX3 [59]. After 45 days, the antibody in serum was collected and purified to 4.8 mg/mL with 90+% purity (Beijing Protein Innovation, China), and the specificity of the antibody was evaluated (Additional file 1: Fig. S7). For protein analysis, the whole body of a larval beetle was homogenized, then lysated in RIPA Lysis Buffer (Beyotime, China) with 1% proteinase inhibitor cocktail (Thermo Scientific, USA) on ice for 2 h. The protein concentration was measured by BCA Protein Assay Kit (Thermo Scientific). After denaturation in boiling water for 5 min, the proteins were separated using Mini-Protean TGX (Bio-Rad, USA) and transferred onto polyvinylidene difluoride membranes (Millipore). Membranes were blocked for non-specific binding using blocking buffer (Thermo Scientific), then incubated with the primary antibody (rabbit anti-ACOX1 serum or rabbit anti-a-TUBULIN, 1:5000) in blocking reagents at room temperature for 2 h. After incubation, the membranes were washed using TBST three times (15 min/each). The washed membranes were incubated with secondary antibody (anti-rabbit IgG, CST, 1:10,000) for 2 h at room temperature and were washed again. Pierce ECL Western blotting Substrate (Thermo Scientific) was added onto the membranes, and the protein stripes were imaged and analyzed on a gel image analyzing system. Three replicates were used for each treatment. The original Western blot figure for each experiment was stored in Additional file 1: Fig. S8.

### Injection of dsRNAs, antagomir, and agomir

Double-stranded RNAs (dsRNAs) were synthesized using primers ligated with T7 RNA polymerase promoter sequences (Additional file 3: Dataset S7, S8) following the protocol of the T7 RiboMax Express RNAi System Kit (Promega, Madison, USA). Twenty larval beetles were injected with dsAcox1 or dsGFP (control) every 72 h, respectively. All the injections were performed using a 7000-series modified microliter syringe (Hamilton, Bonaduz, Switzerland) with 30 μg of dsRNA into the intersegmental membrane between the third and fourth abdominal segments, where the dsRNAs could be transferred to the target genes through the hemolymph. After rearing larvae for 7 days at standard conditions, effectiveness of the knock-down was evaluated by qPCR and Western blot (Additional file 1: Fig. S7), and the amount of ascaroside (asc-C9) and SCPs were also measured.

Antagomir-31-5p and agomir-31-5p are both chemically modified, cholesterol-conjugated, single-strand RNA analogs (Riobio, Guangzhou, China). In vivo delivery of antagomir-31-5p whose sequence is reverse complementary to mature miRNA, efficiently and specifically silences endogenous miRNA, whereas agomir-31-5p could cause the “overexpression” of endogenous miRNA [31]. The sequence of a *C. elegans* miRNA, cel-miR-67-3p (5′ to 3′: UCA CAA CCU CCU AGA AAG AGU AGA), was used as the negative control of antagomir or agomir (antagomir-control or agomir-control). Twenty last-instar larval beetles reared at 25 °C were injected twice as described in dsRNA injection with 500 nM antagomir, agomir, or their controls. At 7 days post-injection, the functions were validated by qPCR for miRNA and mRNA expression, Western blot for protein expression, chemical analysis for ascaroside changes, and SCP measurements for cold hardiness.

To determine the effect of miR-31-5p-*acox1* axis on the SCPs and LT_50_ of beetles, we performed rescue experiments. We first injected the beetles with antagomir-31-5p or antagomir-control, and then rescued the antagomir-31-5p-treated beetles by RNAi by injecting dsAcox1 or exposing them to experimental low-temperature acclimation at 4 °C (AC) for 7 days. Finally, the SCPs were measured and compared between beetles from control, antagomir-31-5p, dsAcox1, and AC groups. Eleven to seventeen replicates were used for each treatment.

### Extraction and analysis of ascaroside

Ascarosides from beetles and nematodes were extracted as previously described [23, 32, 60] with modification. For the larval beetles, it was first confirmed that nematodes were absent from the beetle larvae as determined through microscopic observation. To extract the surface ascarosides, the whole body of each beetle was extracted for 30 min in 1 ml of ethanol [23]. The extracts were purified through filter paper, lyophilized in a vacuum centrifugal concentrator (RVC2-18CD, Sigma), and resuspended in 150 μL of 50% (vol/vol) methanol in water. Subsequently, the liquids were transferred into a liquid chromatography–mass spectrometry (LC–MS) vial and stored at 4 °C until chemical analysis. There were eight replicates for each experiment.

To get the exo-metabolome extracts of *C. elegans*, 100 ml of liquid cultures inoculated with 10,000 mixed stage nematodes was kept at 20 °C and 150 rpm. Concentrated *Escherichia coli* OP50 bacteria pellet from an overnight culture in Lysogeny Broth (LB) medium at 37 °C and 170 rpm was provided as food. After 6 days, the liquid cultures were centrifuged at 800*g* for 2 min. After removing the worms at the bottom, the supernatant was centrifuged again at 2700*g* for 10 min and the harvested supernatants were filtered, lyophilized, and extracted in methanol for 12 h. The extracts were again filtered and lyophilized, then reconstituted in 150 μL of 50% (vol/vol) methanol for HPLC-MS analysis.

The ascaroside analysis was performed using an Agilent 1290 Infinity LC/6460 triple quadrupole MS, equipped with a ZORBAX SB-Aq column (2.1 × 100 mm, 3.5 μm, Agilent, USA) following the previous methods [23]. Asc-C9 was monitored in the negative ion mode (the characteristic product ion monitored was m/z 73 and m/z 303.2). The concentration was then determined using standard curves. Asc-C9 was synthesized following the methods of Zhao et al. [23].

### In vitro luciferase reporter gene assays

The 429-bp sequence (Additional file 3: Dataset S8) of CDS surrounding the predicted miR-31-5p target sites (CACTAGGTTGATTTTTCTTGCCG) was cloned into the psiCHECK-2 vector (Promega, Madison, USA) using an in-fusion cloning system with overlapping primers (Additional file 3: Dataset S7). The fragments of plasmid vector and gene (cDNA) were amplified using Phusion High-Fidelity DNA polymerase (NEB) in a 50 μl reaction, using the following procedure: 98 °C for 30 s, 30 cycles at 98 °C for 5 s, 55 °C for 20 s, and 72 °C for 20 s (the fragments) or 2 min (psiCHECK-2 vector plasmid), then extension at 72 °C for 10 min. The products of vector were treated by *Dpn*I enzyme to digest the remaining circular plasmids. Afterward, the PCR products of psiCHECK-2 vector and CDS were ligated using a Gibson Assembly Cloning Kit (NEB) at 50 °C for 2 h and transfected into DH5*α* competent cells for positive clone screening. Site mutagenesis of the nucleotides complementary to the miR-31-5p seed sequence in the recombinant plasmid was performed using the Fast Mutagenesis System (TransGen, China) with mutation from “TCTTGCC” to “CACCAAA.” All of the recombinant plasmids were extracted using a Plasmid Mini Kit (Qiagen, German). Five hundred microliters of 8 × 10^4^ cells/mL^− 1^ *Drosophila* S2 cells were seeded into each well of a 24-well plate with complete Schneider’s *Drosophila* medium. After 24 h, *Drosophila* S2 cells were co-transfected with 0.4 μg of the luciferase reporter plasmids (WT or MT) and 100 nmol of the agomir-31-5p or agomir-control (Riobio) using 1.5 μL Attractene Transfection Reagent (Qiagen). After 24 h transfection, the activities of the firefly and Renilla luciferases were measured with a Dual-Luciferase Reporter Assay (Promega) using a luminometer (Promega). Eight replicates were done for each treatment.

### RNA immunoprecipitation assay

Polyclonal antibody against AGO1 was developed according to the methods of anti-ACOX1 antibody. The polypeptide used is “DITHPPAGDSRKPSC,” and the specificity of the antibody was also evaluated by Western blot. RIP assay was performed using the Magna RIP Quad kit (Millipore, USA) with the polyclonal antibody against AGO-1 protein (GenScript, Nanjing, China) and a negative control IgG (Cell Signaling Technology). Specifically, six last-instar larval beetles were injected twice with agomir-31-5p or agomir-control, respectively. Seven days following injections, the beetle was homogenized in ice-cold RIP lysis buffer, then stored at − 80 °C overnight. Next, 50 μL magnetic beads were incubated with 5 μg of antibody (anti-AGO1 or IgG) to form bead–antibody complex. The frozen lysates were thawed and centrifuged, and the supernatant was divided into three equal parts. Two parts of which were incubated with the bead–antibody complex (anti-AGO1 or IgG) at 4 °C overnight, and the last one considered as “input” was stored at − 80 °C until RNA extraction. Subsequently, the RNA was extracted by Trizol reagent (Life Technology) and reverse-transcribed into cDNA using the high-capacity RNA-to-cDNA Kit (Applied Biosystems). The precipitated mRNA of *acox1* from both agomir-31-5p- and agomir-control-treated lysates were quantified by qPCR. The IgG control and “input” were assayed to normalize the relative expression and ensure the specificity of the RNA–protein interactions. Six replicates were used for each treatment.

### Heterologous expression of Mal-ACOX1 in *C. elegans*

To validate the role that Mal-*acox1* plays in the regulation of asc-C9, the amount of ascarosides were compared among *C. elegans* wild type strain N2 (Bristol), peroxisomal β-oxidation mutants *acox1* (ok2257) I strain VC1785, and the rescue type strains (*acox1* (ok2257) :: Cel-p:: Mal-*acox1*) with heterologous expression of Mal-ACOX1 in *C. elegans*. Eight replicates were used for each treatment.

To get the rescue strains, the promoter of Cel-*acox1* (pCel) and CDS of Ma-*acox1* were cloned into the pPD95_77 plasmid vector using the in-fusion cloning system (Additional file 3: Dataset S7, S8). The recombinant plasmids were injected into the gonads of mutant *acox1* (ok2257) I strain VC1785 with the green fluorescent protein (GFP) plasmids. F2 and subsequent offspring with GFP were screened by a fluorescent microscope. Finally, two lines (line 1 and line 2) were selected for ascaroside analysis.

### Ascaroside feeding experiment

To test the effect of ascaroside on beetle’s SCPs and mortality, ascaroside feeding experiment was conducted as previously described [23]. Four hundred microliters of asc-C9, asc-△C6 (negative control), or sterilized water (control) was added into the artificial diets (200 g of sawdust and 15 g of agar were added to 550 ml of distilled water and mixed, and 400 μl of asc-C9 at 0.1 nM was added). As previously described, asc-C9 and asc-△C6 were synthesized and tested at 0.1 nM and 3.97 nM, respectively [23]. Then, larval beetles were reared on ascaroside added or control diets in a climate chamber at a 12:12 h light:dark (L:D) cycle at 25 °C. Twenty individuals were sampled for SCP measurement from each treatment every day for 10 days. Samples from the 10th day were used for qPCR and mortality tests. Six and three replicates were used for each treatment in qPCR and mortality tests, respectively.

### Statistical analyses

For mortality experiments, a probit regression was used to calculate LT_50_ values and differences were compared by extra sum-of-squares *F* test. Data analyses were performed using GraphPad Prism (version 6.01) (GraphPad software, Inc., San Diego, CA, USA) [61]. For other experiments, Levene’s test was used to test the assumption of homoscedasticity. Student’s *t* test was used for two-group comparisons. One-way ANOVA with Tukey’s HSD multiple comparison was performed for more than two-group comparisons. All statistical analyses were performed by IBM SPSS 18.0 software. Differences were considered significant at *P* < 0.05.

## Supplementary information

**Additional file 1: Figure S1.** The statistics of miRNAs. **Figure S2.** Validation of the expression of down-regulated miRNAs after cold acclimation by qPCR. **Figure S3.** Phylogenetic tree of ACOXs and the predicted sites of Mal-miR-31-5p. **Figure S4.** The amount of asc-C9 after cold acclimation in both laboratory and field. **Figure S5.** The effect of Asc-△C6 (negative control) on beetle cold hardiness. **Figure S6.** The expression of cryoprotectants related genes after asc-C9 treatment. **Figure S7.** The expression of mRNA and protein of acox1 after RNAi. **Figure S8.** The original western blot figures in this study.

**Additional file 2: Table S1.** The sequences of significantly differently expressed miRNAs after cold acclimation. **Table S2.** The expression of the up-regulated miRNAs after cold acclimation through deep sequencing. **Table S3.** GenBank accession numbers for genes used in this study.

**Additional file 3: Dataset S1.** Differently expressed transcripts (DETs) after low temperature acclimation. **Dataset S2.** KEGG enrichment of DETs after low temperature acclimation (*P* < 0.05). **Dataset S3.** Differently expressed transcripts after feeding asc-C9. **Dataset S4.** Enriched genes in metabolic process by GO analysis. **Dataset S5.** The enrichment GO terms of differently expressed transcripts (DETs) after feeding asc-C9. **Dataset S6.** The programs used in bioinformatic analysis in this study. **Dataset S7.** Primers used in this study. **Dataset S8.** The nucleotide sequences used in this study.

## Data Availability

All data generated or analyzed during this study are included in this published article and its supplementary information files.
