# Supplementary material for: miR-31-5p regulates cold acclimation of the wood-boring beetle Monochamus alternatus via ascaroside signaling
Source: BMC Biol. 2020 Nov 27;18:184. doi: 10.1186/s12915-020-00926-w (PMC7697373; doi:10.1186/s12915-020-00926-w)
Supplement: Supplementary file 1 — Additional file 1: Figure S1. The statistics of miRNAs. Figure S2. Validation of the expression of down-regulated miRNAs after cold acclimation by qPCR. Figure S3. Phylogenetic tree of ACOXs and the predicted sites of Mal-miR-31-5p. Figure S4. The amount of asc-C9 after cold acclimation in both laboratory and field. Figure S5. The effect of Asc-△C6 (negative control) on beetle cold hardiness. Figure S6. The expression of cryoprotectants related genes after asc-C9 treatment. Figure S7. The expression of mRNA and protein of acox1 after RNAi. Figure S8. The original western blot figures in this study. [file 12915_2020_926_MOESM1_ESM.docx]

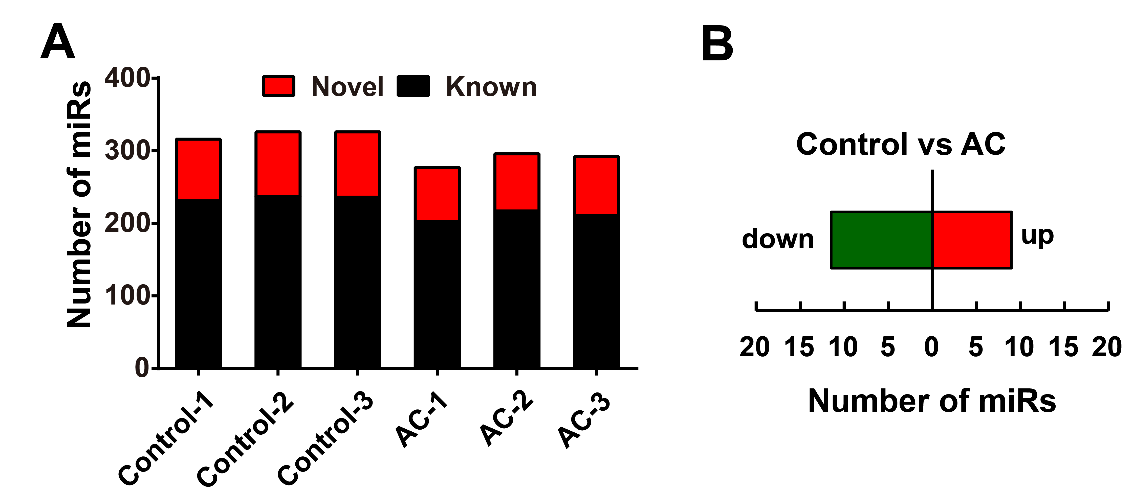


**Fig S1. The statistics of miRNAs.**

**A.** The number of known and novel miRNAs (miRs) in six miRNA libraries of larval beetles. **B.** The number of miRNAs which are up-regulated and down-regulated after low temperature acclimation. Control (25 ℃); AC, low temperature acclimation (4 ℃).


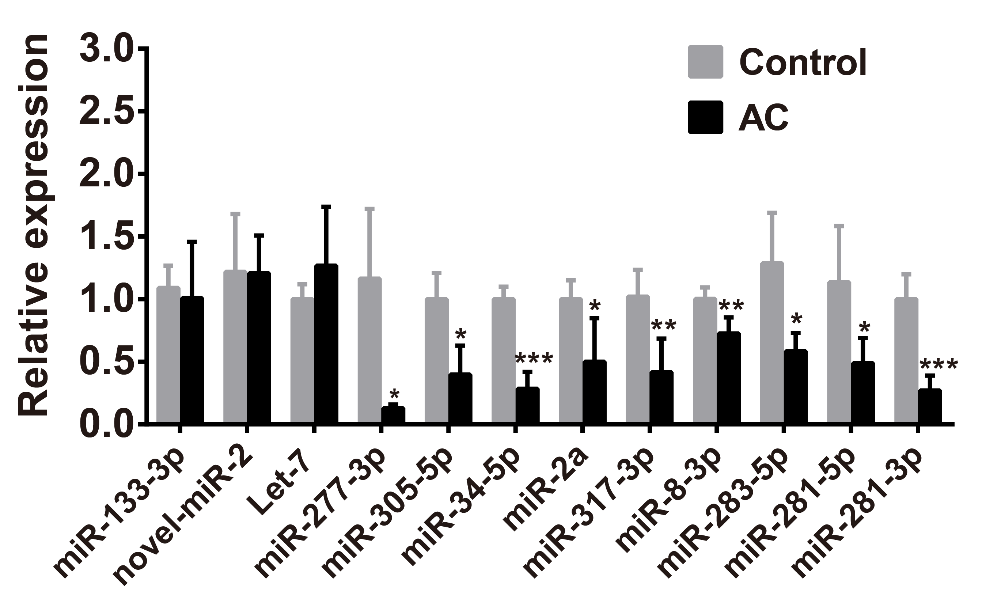


**Fig S2. Validation of the expression of down-regulated miRNAs after cold acclimation by qPCR.**

The data are shown as mean ± s.e.m. Student’s *t*-test with two-tailed was used to test the different significance, **P* < 0.05, ***P* < 0.01, ****P* < 0.001. Control (25 ℃); AC, low temperature acclimation (4 ℃).

**
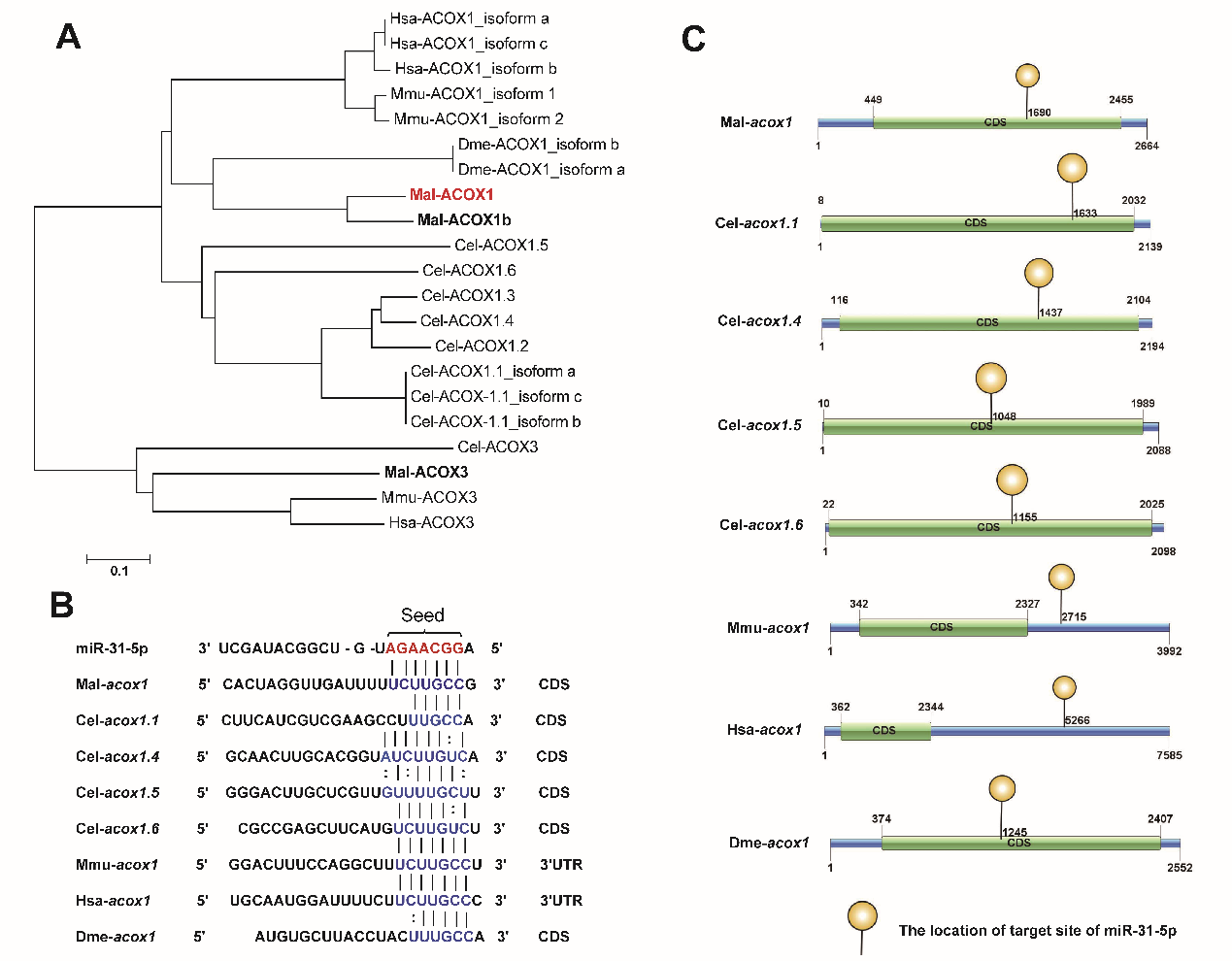
**

**Fig S3. Phylogenetic tree of ACOXs and the predicted sites of Mal-miR-31-5p.**

**A.** Maximum-likelihood (ML) phylogeny based on protein sequences of ACOXs from *Homo sapiens* (Hsa), *Mus musculus* (Mmu), *Drosophila melanogaster* (Dme), *C. elegans* (Cel) and *Monochamus alternatus* (Mal).The ML tree was constructed using Mega 6 with default settings and 1000 bootstrap replicates. Multiple sequence alignment was conducted using ClustalW2.0 with default setting. Mal-ACOX1 is a target of miR-31-5p in *M. alternatus*. **B.** The putative target sites of Mal-miR-31-5p in *acox1* genes from different animals using RNAhybrid. **C.** The relative position of target site of Mal-miR-31-5p on *acox1* genes in different species. The gene structures were illustrated by IBS 1.0.1.


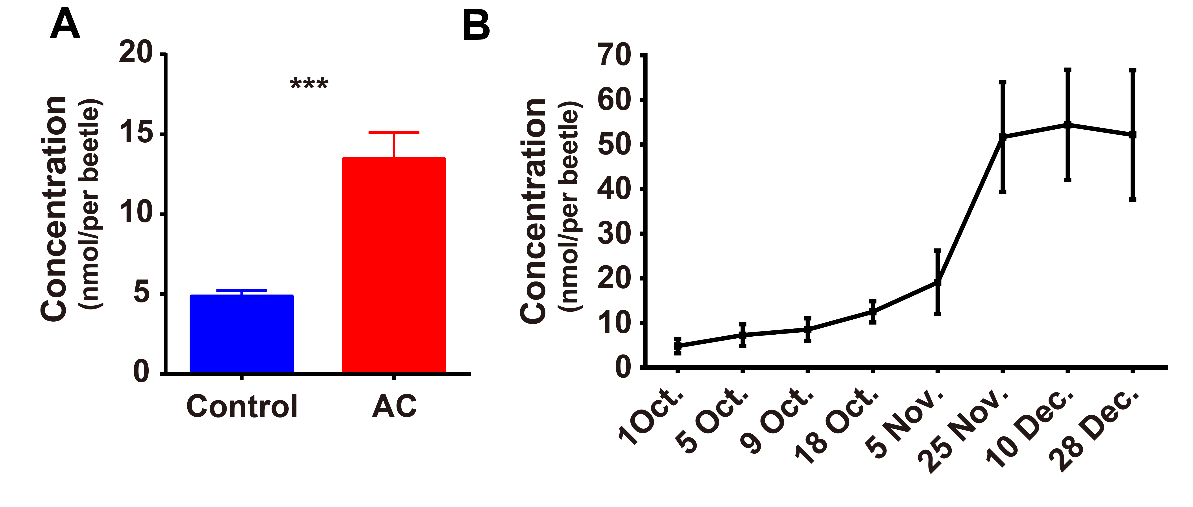


**Fig S4. The amount of asc-C9 after cold acclimation in both laboratory and field.**

**A.** The concentration of asc-C9 in control (25 ℃) and low temperature acclimation (AC, 4 ℃) larval beetles in laboratory (n = 8). Student’s *t* test was used for the significance analysis, ****P* < 0.001. The beetles used in this experiment were collected at 15 Oct. 2017. **B.** The concentration of asc-C9 from October to December in field.


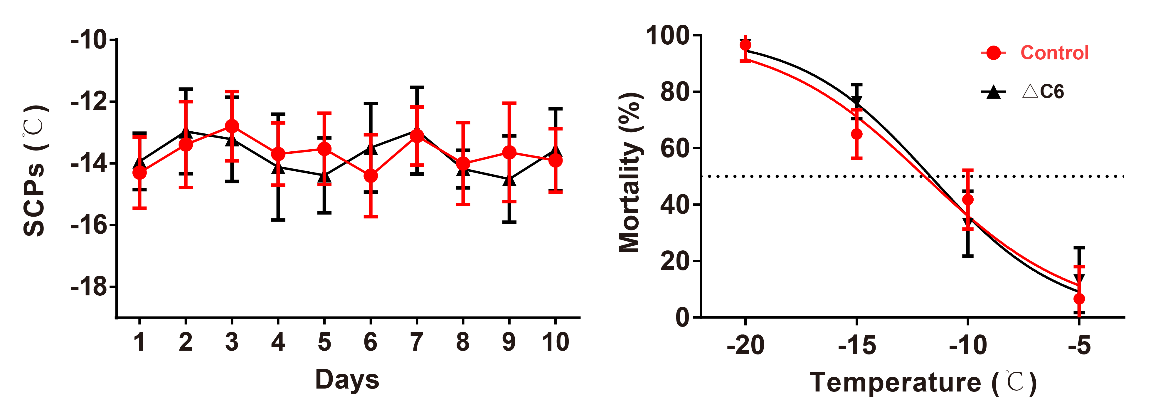


**Fig S5. The effect of Asc-△C6 (negative control) on beetle cold hardiness.**

**A.** The effect of asc-△C6 on the supercooling points (SCPs) of the last instar larval beetles (n = 20). **B.** The effect of asc-△C6 on mortality of the last instar larval beetles (n = 10 for each treatment). Lethal temperature causing 50% mortality (LT50) was determined by probit regression. The data in **A and B** are shown as mean ± s.e.m. Student’s *t*-test with two-tailed was used to test the different significance. △C6, asc-△C6 fed beetles; Control beetles. The last larval beetles used in A and B were collected at 20-25 Oct. 2019.

**
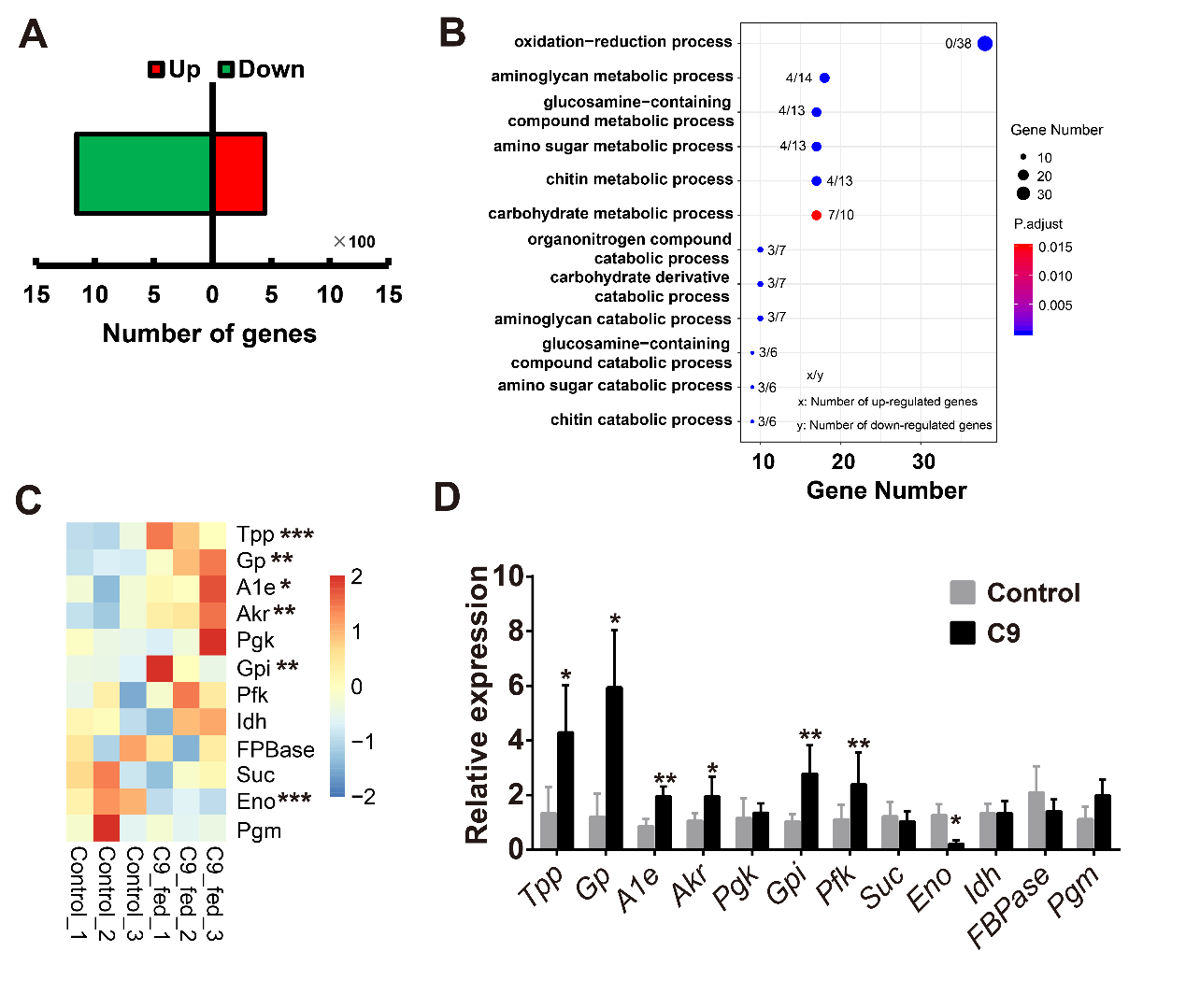
**

**Fig S6. The expression of cryoprotectants related genes after asc-C9 treatment.**

**A.** The number differently expressed genes (DEGs) between control and asc-C9 fed beetles in RNA-seq. **B**. The number of genes in the significantly enriched processes (*P* < 0.05). **C**. Hierarchical clustering of the expression of the genes related to the synthesis and metabolism of these three cryoprotectants based on RNA-seq (n = 3).

**D.** qPCR of the genes in the pathway of the synthesis and metabolism of the three cryoprotectants (n = 6). The data were shown as mean ± s.e.m. Student’s *t*-test with two-tailed was used to test the different significance. **P* < 0.05; ***P* < 0.01; ****P* < 0.001. Genes legends: *Tpp*, trehalose-phosphate phosphatase; *Gp*, glycogen phosphorylase; *A1e*, aldose-1-epimerase; *Akr*, aldo-keto reductase; *Pgk*, phosphoglycerate kinase; *Gpi*, glucose-6-phosphate isomerase; *Pfk*, 6-phospho-fructokinase; *Suc*, succinyl-CoA ligase; *Eno*, enolase; *Idh*, isocitrate dehydrogenase; *FBPase*, fructose-1,6-bisphosphatase; *Pgm*, phosphoglycerate mutase; G1P, glucose-1-phosphate; GAP, glyceraldehyde-3-phosphate.

**
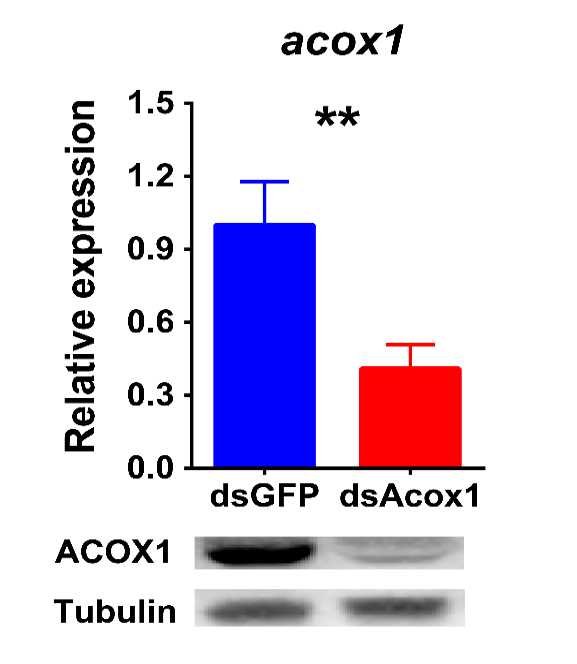
**

**Fig S7. The expression of mRNA and protein of *acox1* after RNAi.**


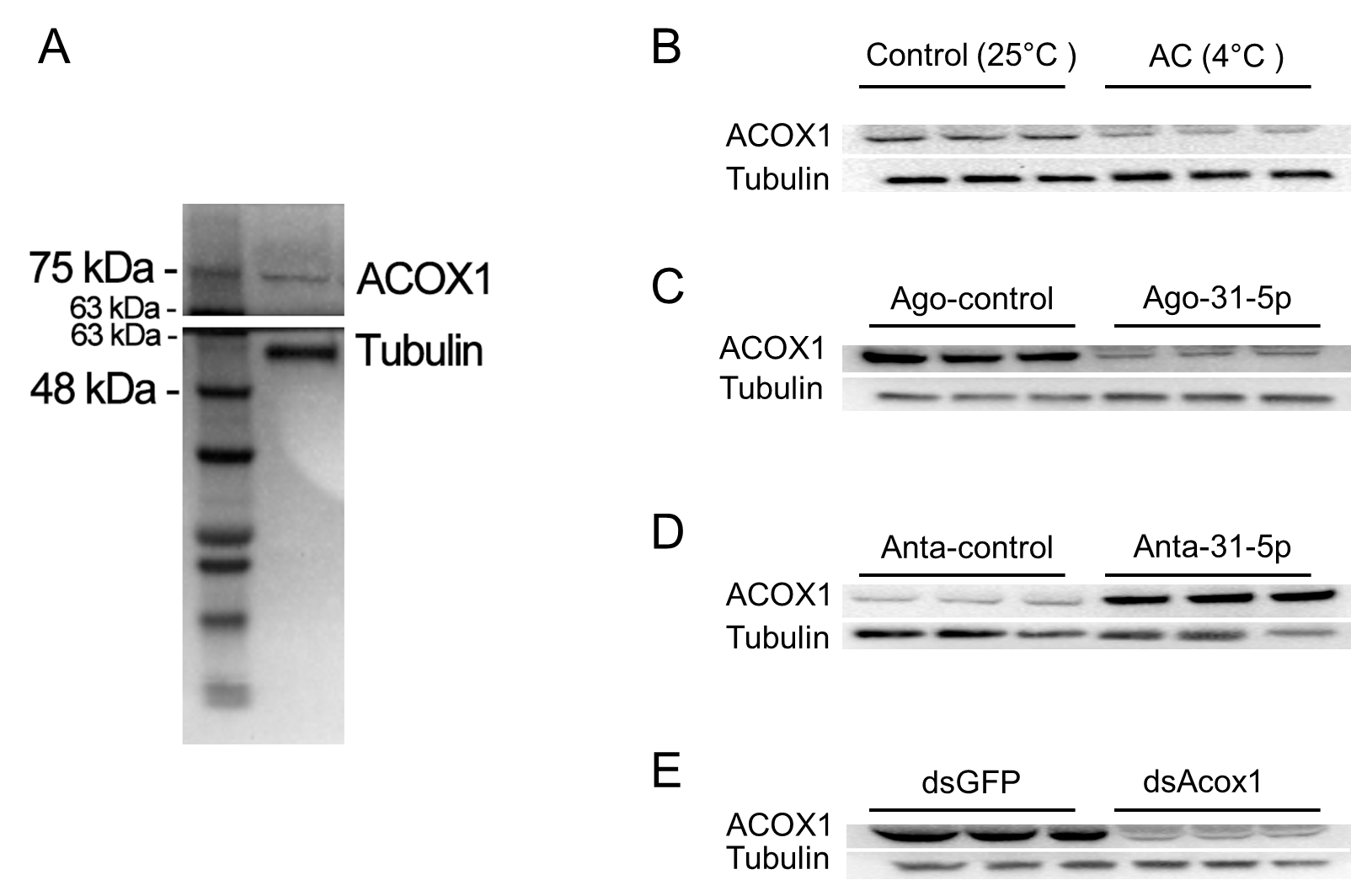


**Fig S8. The original western blot figures in this study.**

**A.** The size of ACOX1 (about 75 kDa) and Tubulin (about 56 kDa) in *M. alternatus*. **B, C, D and E**. The original western blot figure of ACOX1 and Tubulin after cold acclimation (4 °C) (B), injection of agomir-31-5p (C) and antagomir-31-5p (D), and RNAi (E). They were cropped and correspond to Figure 4A, 4B, 4C and Figure S7, respectively. The last larval beetles used in A, B and D were all collected at 10-15 Nov. 2017, while beetles in C and E were collected at 5 Oct. 2017.
