# Supplementary material for: miR-31-5p regulates cold acclimation of the wood-boring beetle Monochamus alternatus via ascaroside signaling
Source: BMC Biol. 2020 Nov 27;18:184. doi: 10.1186/s12915-020-00926-w (PMC7697373; doi:10.1186/s12915-020-00926-w)
Supplement: Supplementary file 2 — Additional file 2: Table S1. The sequences of significantly differently expressed miRNAs after cold acclimation. Table S2. The expression of the up-regulated miRNAs after cold acclimation through deep sequencing. Table S3. GenBank accession numbers for genes used in this study. [file 12915_2020_926_MOESM2_ESM.docx]

**Table S1. The sequences of significantly differently expressed miRNAs after cold acclimation.**

| **miRs** | **Sequence** |
| --- | --- |
| let-7 | UGAGGUAGUUGGUUGUAUAGU |
| miR-10-3p | CAAAUUCGGUUCUAGAGAGGU |
| miR-133-3p | UUGGUCCCCUUCAACCAGCUGU |
| miR-184-3p | UGGACGGAGAACUGAUAAGGG |
| miR-190-5p | AGAUAUGUUUGAUAUUCUUGG |
| miR-277-3p | UAAAUGCACUAUCUGGUACGACA |
| miR-281-5p | AAGAGAGCUAUCCGUCGACAGU |
| miR-281-3p | ACUGUCAUGGAGUUGCUCUCUU |
| miR-283-5p | AAAUAUCAGCUGGUAAUUCUGGG |
| miR-2a | UAUCACAGCCAGCUUUGAUGAGCG |
| miR-305-5p | AUUGUACUUCAUCAGGUGCUCUG |
| miR-31-5p | AGGCAAGAUGUCGGCAUAGCU |
| miR-317-3p | UGAACACAGCUGGUGGUAUCUCAG |
| miR-34-5p | UGGCAGUGUGGUUAGCUGGUUGUG |
| miR-71 | UGAAAGACAUGGGUAGUGAGAUG |
| miR-7-5p | UGGAAGACUAGUGAUUUUGUU |
| miR-8-3p | UAAUACUGUCAGGUAAAGAUGUC |
| miR-9a-5p | UCUUUGGUUAUCUAGCUGUAU |
| novel-miR-1 | ACGUAUACUGAAUGUAUCCUGA |
| novel-miR-2 | CCAGAUCUAACUCUUCCAUGCGA |

**Table S2. The expression of the up-regulated miRNAs after cold acclimation through deep sequencing.**

| **miRNA** | **25℃ (rpm ± sd)** | **4℃ (rpm ± sd)** | **Foldchange (AC/CK)*^a^*** | **P-value** | **FDR** |
| --- | --- | --- | --- | --- | --- |
| miR-184-3p | 1414.78± 219.32 | 2382.71 ± 169.58 | 1.3076 | 0.0006 | 0.0103 |
| novel-miR-1 | 8.67 ± 3.03 | 16.61 ± 1.84 | 1.9163 | 0.0009 | 0.0289 |
| miR-71 | 590.69 ± 5.63 | 880.43 ± 102.09 | 1.4905 | 0.0000 | 0.0001 |
| **miR-31-5p*^b^*** | **1369.30 ± 216.53** | **3984.73 ± 143.16** | **2.9101** | **0.0001** | **0.0000** |
| miR-7-5p | 65.36 ± 9.37 | 162.77 ± 13.51 | 2.4902 | 0.0000 | 0.0000 |
| miR-190-5p | 21.33 ± 0.54 | 27.16 ± 8.07 | 1.2732 | 0.0059 | 0.0255 |
| miR-10-3p | 74.42 ± 16.41 | 99.13 ± 9.67 | 1.3321 | 0.0014 | 0.0196 |
| miR-9a-5p | 923.87 ± 106.60 | 1049.15 ± 190.42 | 1.1356 | 0.0026 | 0.0302 |

***^a^*** AC, low temperature acclimation (4 ℃); CK, control (25 ℃).

***^b^*** miR-31-5p exhibited the greatest extent of expression up-regulation as well as the highest abundance.

**Table S3. GenBank accession numbers for genes used in this study.**

| **Genes** | **Accession numbers** |
| --- | --- |
| *Tpp* | MK531792 |
| *Gp* | MK531793 |
| *A1e* | MK531794 |
| *Akr* | MK531795 |
| *Pgk* | MK531796 |
| *Gpi* | MK531797 |
| *Pfk* | MK531798 |
| *Suc* | MK531799 |
| *Eno* | MK531800 |
| *Idh* | MK531801 |
| *FBPase* | MK531802 |
| *Pgm* | MK531803 |
| *Amt* | MK531804 |
| *Hairy* | MK531805 |
| *α-actinin* | MK531806 |
| *Acox1* | MK531807 |
| *Pdh* | MK531808 |
| *Ago1* | MW115942 |
